# Overview of Therapeutic Ultrasound Applications and Safety Considerations: 2024 Update

**DOI:** 10.1002/jum.16611

**Published:** 2024-11-11

**Authors:** Kenneth B. Bader, Frederic Padilla, Kevin J. Haworth, Nicholas Ellens, Diane Dalecki, Douglas L. Miller, Keith A. Wear, Bioeffects Committee of the American Institute of Ultrasound in Medicine, Kenneth Bader, Jason T. Nomura, Aiguo Han, Alfred C.H. Yu, Alycen Wiacek, Diane Dalecki, Douglas L. Miller, Kevin J. Haworth, Holly S Lay, Kenneth Hoyt, Inder Raj Singh Makin, Jacques S. Abramowicz, Marie Muller, Siddhartha Sikdar, Zheng Feng Lu, Mitra Aliabouzar, Corinne E Wessner, J. Brian Fowlkes, Gerald R. Harris, Jennifer E. Bagley, John Donlon, Keith A. Wear, Kurt Sandstrom, Paul L. Carson, Felicia M. Toreno, Frederic Padilla, Kevin J. Haworth, Douglas L. Miller, Wayne Moore, Jacques S. Abramowicz, Jennifer E. Bagley, Keith A. Wear, Shahram Vaezy

**Affiliations:** ^1^ Department of Radiology University of Chicago Chicago Illinois USA; ^2^ Gene Therapy Program Focused Ultrasound Foundation Charlottesville Virginia USA; ^3^ Department of Radiology University of Virginia Health System Charlottesville Virginia USA; ^4^ Department of Pediatrics University of Cincinnati Cincinnati Ohio United States; ^5^ Department of Internal Medicine University of Cincinnati Cincinnati Ohio USA; ^6^ Department of Biomedical Engineering University of Cincinnati Cincinnati Ohio USA; ^7^ Alpheus Medical, Inc. Chanhassen Minnesota USA; ^8^ Department of Biomedical Engineering University of Rochester Rochester New York USA; ^9^ Department of Radiology University of Michigan Health System Ann Arbor Michigan USA; ^10^ Center for Devices and Radiological Health U.S. Food and Drug Administration Silver Spring Maryland USA

**Keywords:** bioeffects, image‐guided therapy, therapeutic ultrasound

## Abstract

A 2012 review of therapeutic ultrasound was published to educate researchers and physicians on potential applications and concerns for unintended bioeffects (doi: 10.7863/jum.2012.31.4.623). This review serves as an update to the parent article, highlighting advances in therapeutic ultrasound over the past 12 years. In addition to general mechanisms for bioeffects produced by therapeutic ultrasound, current applications, and the pre‐clinical and clinical stages are outlined. An overview is provided for image guidance methods to monitor and assess treatment progress. Finally, other topics relevant for the translation of therapeutic ultrasound are discussed, including computational modeling, tissue‐mimicking phantoms, and quality assurance protocols.

**Abbreviations**
AAPM: American Association of Physicists in Medicine
ASMase: acidic sphingomyelinase
BBB: blood–brain barrier
BSCB: blood–spinal cord barrier
CAR‐T cells: chimeric antigen receptor T cells
CMS: U.S. Centers for Medicare and Medicaid Services
CT: computerized tomography
DAMP: damage‐associated molecular patterns
EMA: European Medicines Agency
FDA: U.S. Food and Drug Administration
IEC: International Electrotechnical Commission
LIPUS: low‐intensity pulsed ultrasound
MRI: magnetic resonance imaging
NOR‐SASS: Norwegian Sonothrombolysis in Acute Stroke Study
QA: quality assurance
TMM: tissue‐mimicking material
US: ultrasound

Ultrasound is known commonly as a diagnostic imaging modality that has an excellent safety record when used as intended by a qualified sonographer.^1^ Although the mechanical energy of the ultrasound wave is nonionizing, it can still induce biological effects for sufficient exposure conditions.^2^ Most diagnostic ultrasound imaging systems display indices to help the sonographer understand the likelihood of bioeffects, and avoid unnecessary exposure.^3^, ^4^ It is also possible to harness ultrasound to cause bioeffects intentionally for therapeutic purposes.^5^ At the time this statement was written (2024), multiple therapeutic ultrasound devices have been approved, cleared, or had de novo request granted by the U.S. Food and Drug Administration (FDA) since 2012 (Table 1), including applications in thermal or mechanical ablation of pathologic tissue, or to enhance the delivery of therapeutic drugs. Further, the U.S. Centers for Medicare and Medicaid Services (CMS) provided reimbursement codes for ultrasound treatments of certain thrombotic embolisms (37211), palliation of pain due to bone metastases (C9734), essential tremor (0398T), tremor‐dominant Parkinson's disease (0398T), prostate conditions (55880 and C9734), solid renal tumors (C9790), and primary and metastatic liver tumors (0686T). The European Medicines Agency (EMA) approved devices for these applications and others, including hypertension,^6^ varicose veins,^7^ thyroid nodules,^8^ primary and metastatic tumors (pancreas,^9^ soft tissue,^10^ and breast^11^), along with other musculoskeletal^12^, ^13^ and neurological applications.^14^, ^15^ It is anticipated that new applications and devices will be approved or cleared over time. Beyond these approved and cleared indications for use, more than 1,900 active investigations with therapeutic ultrasound were listed on clinicaltrials.gov, ranging from Phase I safety‐focused investigations to Phase III efficacy‐focused studies (search terms: “therapeutic ultrasound,” “focused ultrasound,” “HIFU” for high‐intensity focused ultrasound, “HITU” for high‐intensity therapeutic ultrasound, search date August 1, 2024).

**Table 1:** Therapeutic Ultrasound Devices Approved, Cleared, or Have Had a De Novo Request Granted by the FDA Since 2012

Year (# Devices)	Therapy Method	Therapeutic Outcome	Bioeffect Mechanism	Device Characteristics			General Reference
				Applicator	Frequency	Pulse Type	
2012–2023 (>20)	Surgical tools	Tissue cutting; fragmentation; emulsification; vessel sealing	Mechanical	Handheld	22–55 kHz	CW	Schafer, 2023
2013–2023 (12)	Phacoemulsification	Fragmentation and removal of cataracts	Vibration, cavitation	Generator with probe	~40 kHz	CW	Packer, 2005
2013–2022 (10)	Physiotherapy	Pain relief, treatment of muscle spasms and joint contractures	Thermal	Handheld	90 kHz (1), 1–3 MHz (9)	PW, CW	Watson, 2008
2012–2021 (5)	Extra‐corporeal shock wave lithotripsy	Fragmentation of calculi in kidney and/or ureter, and/or pancreas	Compressive and tensile stress, cavitation	Mainframe with X‐ray and/or US monitoring	Shock waves	SW	Weizer 2007
2012–2020 (4)	Intra‐corporeal lithotripsy	Fragmentation of calculi in kidney, ureter, and/or urinary bladder	Compressive and tensile stress	Per‐cutaneous probes	~20–24 kHz	CW, PW	Lowe, 2009
2021 (1)	Intra‐vascular lithotripsy	Low‐pressure balloon dilatation of calcified, stenotic de novo coronary arteries prior to stenting	Mechanical	Catheter‐based probe; angiographic monitoring	500 kHz	PW	Neleman 2023
2014 (1)	Low‐power intra‐vascular ultrasound	Facilitation of delivery of thrombolytic agents	Micro‐streaming	Catheter with 6–30 transducers; angiography monitoring	2–2.5 MHz	Modulated PW	Tachibana 1992
2012 (1)	HIFU	Pain palliation of metastatic bone cancer	Thermal	Phased array	0.5–1.35 MHz	8–20 second burst	Napoli 2013
2013 (1)	HIFU	Disruption of subcutaneous adipose tissue	Thermal	Handheld	2 MHz	PW	Bader 2021
2013 (2)	HIFU	Skin tightening, wrinkle reduction, eyebrow lift	Thermal	Handheld; Sometimes US monitoring	4–12 MHz	PW	Bader 2021
2013 (1)	HIFU	Disruption of subcutaneous adipose tissue	Mechanical	Handheld	200 kHz	PW	Bader 2021
2015 (1)	HIFU	Ablation of prostatic tissue	Thermal	Transrectal dual‐sided transducer; US monitoring	4 MHz	3 second burst	Sanghvi, 2017
2015 (1)	HIFU	Ablation of prostatic tissue	Thermal	Transrectal curved array; US monitoring	3 MHz	6 second burst	Warmuth 2010
2019 (1)	HIFU	Ablation of prostatic tissue	Thermal	Transurethral linear array; MRT monitoring	4–4.8 MHz and 13.4–14.4 MHz	CW	Kotz 2021
2021 (1)	HIFU	Ablation of prostatic tissue	Thermal	Endorectal phased array; MRT monitoring	2.3 MHz	Up to physician	Napoli 2013
2016 (1)	HIFU	Thalamotomy treatment of idiopathic Essential Tremor	Thermal	Transcranial array; MRT monitoring	~650 kHz	~20 second burst	Elias 2013
2018 (1)	HIFU	Thalamotomy treatment of tremor‐dominant Parkinson's disease	Thermal	Transcranial array; MRT monitoring	~650 kHz	~20 second burst	Bond, 2017
2020 (1)	HIFU	Treatment of osteoid osteoma	Thermal	Phased array	1.2 MHz	16–48 second burst	Yarmolenko 2018
2021 (1)	HIFU	Pallidotomy treatment of idiopathic Parkinson's disease	Thermal	Transcranial array; MRT monitoring	~650 kHz	~20 second burst	Eisenberg, 2020
2022	HIFU	Bilateral thalamotomy treatment of idiopathic essential tremor	Thermal	Transcranial array; MRT monitoring	~650 kHz	~20 second burst	Iorio‐Morin, 2021
2022 (1)	LIPUS	Bone fracture healing	Unknown	Wearable	1.5 MHz	200 microsecond burst	Padilla, 2014
2023 (1)	HIFU	Non‐invasive destruction of liver tumors	Mechanical	Extra‐corporeal array transducer	683 kHz	PW	Wah, 2023
2023 (1)	HIFU	Renal denervation to treat hypertension	Thermal	Catheter with US transducer	9 MHz	7 second burst	Daemen, 2015

Numbers in parentheses indicate numbers of devices included in that row. For rows with multiple devices, entries are typical but may not encompass the full range of device capabilities. MRT, magnetic resonance thermometry; US, ultrasound (imaging); HIFU, high‐intensity focused ultrasound; LIPUS, low‐intensity pulsed ultrasound; CW, continuous wave; PW, pulsed wave; SW, shock wave.

The field of therapeutic ultrasound has been reviewed extensively, notably by members of the Bioeffects Committee of the American Institute of Ultrasound in Medicine in 2012.^5^ Since that time, the field of therapeutic ultrasound has expanded to new fronts in technology, translational applications, and clinical implementation. The goal of this review is to provide an update on the field from 2012 to 2024. In addition to clinical applications, the topics of image guidance, challenges to the field, and other considerations (eg, tissue phantoms, quality assurance, simulations, reporting acoustic parameters, and patient safety) are addressed.

## Basis for Therapeutic Ultrasound Applications

**Figure 1:**
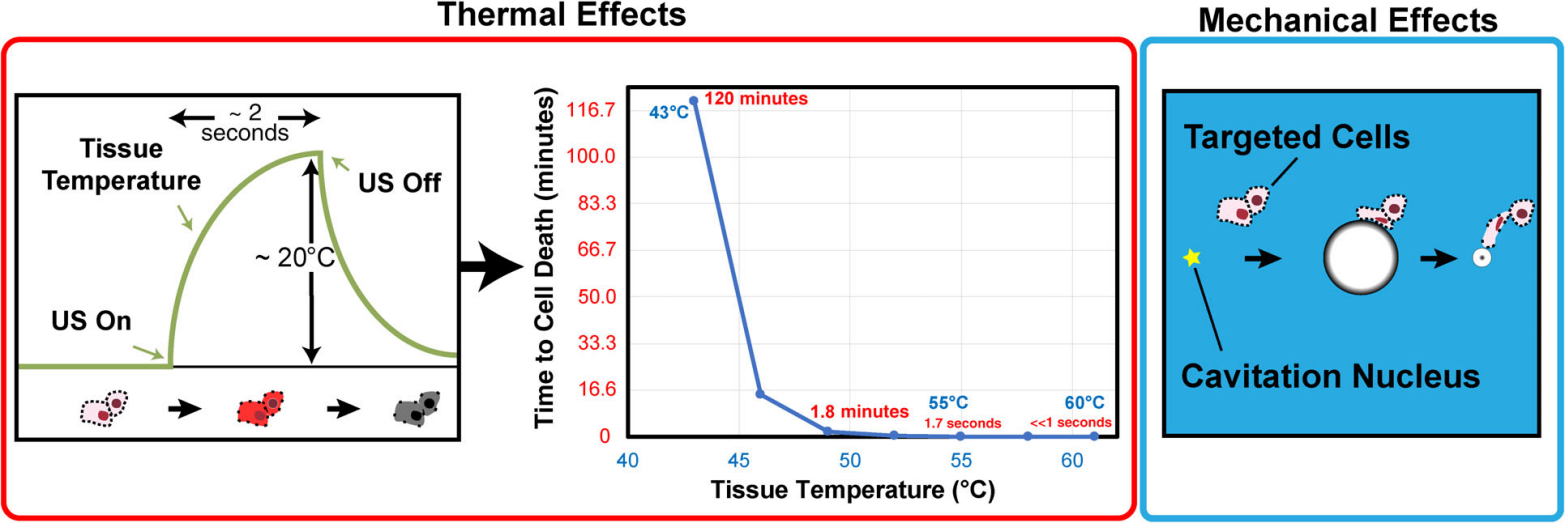
(Left) Absorption of the ultrasound (US) energy heats tissue within the focal zone. Bioeffects that range from mild hyperthermia to thermal necrosis or boiling can be induced due to heating. (Middle) Schematic of temperature–time isodamage relationship leading to irreversible cell death as a thermal bioeffect. Cavitation and heating are not mutually exclusive effects. Cavitation can alter the rate of heating, and temperature elevation can reduce the cavitation threshold pressure. (Right) Schematic of bubble oscillations in response to ultrasound. Cavitation is capable of producing significant mechanical bioeffects, including cavitation, ablation, sonoporation, and streaming. Time increases from left to right as a cavitation nucleus under the influence of ultrasound grows into a microbubble that expands to a maximum diameter and then collapses. Damage can be imparted to cells surrounding the bubble during these oscillations.

There are 2 known physical mechanisms caused by ultrasound that result in bioeffects: thermal and mechanical interactions (Figure 1).^16^ Heating occurs as ultrasound energy is absorbed by the tissue.^17^ The likelihood of cell death due to heating can be gauged by thermal dose, which combines information about the degree and duration of temperature rise in tissue.^18^ The transfer of momentum from the ultrasound wave to tissue or biofluids can cause mechanical displacement or fluid streaming respectively, each of which are known mechanisms of mechanical bioeffects.^19^ Cavitation is another source for mechanical bioeffects, and is caused through interactions between the ultrasound pulse and gaseous nuclei such as microbubble contrast agents.^20^, ^21^ The behavior of cavitation is categorized by 2 primary descriptors: inertial or stable.^22^ Inertial cavitation often occurs when a microbubble expands more than 2‐fold during the tensile phase of the ultrasound pressure field.^22^ The pressures in the fluid or tissue surrounding the microbubble overcome its internal pressures, resulting in a rapid contraction. The near symmetric collapse generates a large energy density that can damage nearby biological structures.^21^ In contrast, stable cavitation is a lower amplitude, sustained microbubble oscillation that is less likely to cause irreversible mechanical damage to tissue.

Triggering thermal and mechanical bioeffects can result in the desired therapeutic endpoints. Different bioeffects will require different ultrasound exposure conditions. Heating is influenced by frequency, acoustic power, in situ spatial‐peak, time‐average intensity (*I*_*SPTA*_), and tissue attenuation.^23^ Radiation force and fluid streaming that give rise to mechanical bioeffects are driven in part by *I*_*SPTA*_ and frequency.^24^, ^25^ Attenuation and viscosity are also contributing factors for radiation force and streaming, respectively.^26^, ^27^ The peak negative pressure and frequency of the ultrasound pulse are among the primary controlling factors in cavitation.^28^, ^29^ Other factors such as the pulse repetition frequency are also important considerations for cavitation,^30^ and the population of microbubble nuclei.^31^, ^32^ Different bioeffects will require different amounts of ultrasound, and thermal and mechanical mechanisms can occur simultaneously for some exposure conditions. In situ factors also affect bioeffects, including tissue properties (eg, density, sound speed, attenuation coefficient, backscatter coefficient, acoustic impedance, thermal conductivity, perfusion, elasticity, and viscosity), and specifications of the therapeutic device (eg, geometry, frequency, bandwidth, pulse duration, pulse repetition frequency, acoustic intensity, and pressure amplitude).

## Treatment of Tissues Without Exogenous Agents

**Figure 2:**
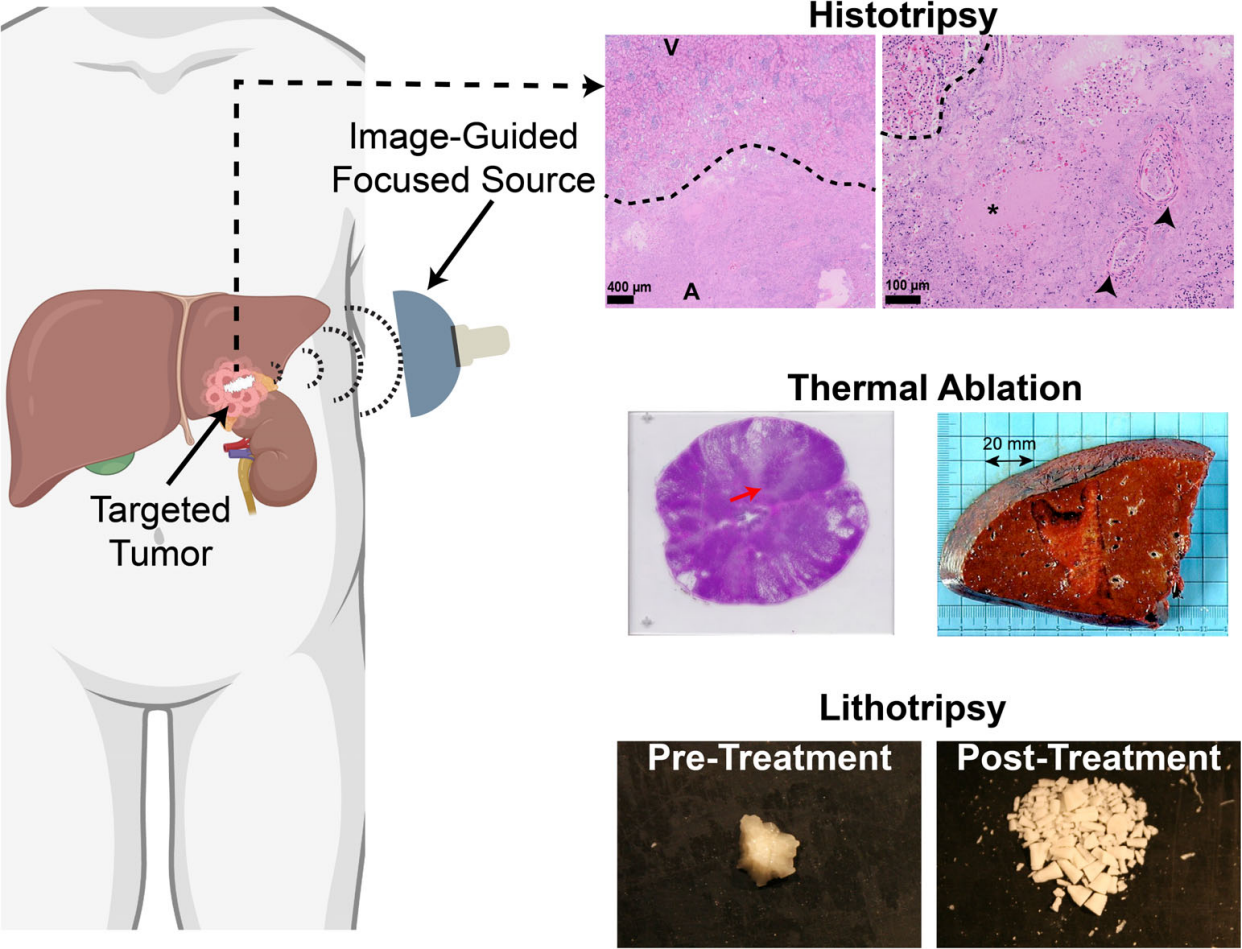
A subset of therapeutic ultrasound applications without exogenous agents. Ultrasound is frequently applied transcutaneously to targets under image guidance, though intravascular sources are also under development. *Histotripsy* uses ultrasound pulses with sufficient tension to generate bubbles in situ that reduce ablated tissue (A) to acellular debris, as noted in the left image. At increased magnification there is a sharp boundary (dashed line) between viable (V) and tissue ablated mechanically (A). Treated regions exhibit viable vessels (arrowheads) and loss of cell nuclei (*) within the treatment zone (Reprinted from *IEEE Trans Biomed Eng*, vol. 71, no. 6, Development of Convolutional Neural Network to Segment Ultrasound Images of Histotripsy Ablation, pp. 1789–1797, Copyright 2024, under creative commons license CC BY‐NC‐ND 4.0). *Thermal Ablation* is achieved through absorption of ultrasound, and has been used to treat patients with pathological conditions in the prostate (left, Reprinted from *Medical Physics*, vol. 46, no. 2, MRI‐guided transurethral insonation of silica‐shell phase‐shift emulsions in the prostate with an advanced navigation platform, pp. 774–788, Copyright 2019, with permission from Wiley) and liver (right, Reprinted from *PLoS ONE*, vol. 10, no. 2, First Clinical Experience of Intra‐Operative High Intensity Focused Ultrasound in Patients with Colorectal Liver Metastases: A Phase I‐IIa Study, article number e0118212, Copyright 2015, reproduced under creative commons license CC BY 4.0). Several forms of *Lithotripsy* have been developed to break down mineralized tissue in the kidney and pancreas and vasculature. Burst wave lithotripsy applies tone bursts with broadly focused ultrasound that is able to comminute multiple types of kidney stones, including the struvite sample displayed above (Images provided courtesy Adam Maxwell). This figure was created in part in BioRender.com

In the absence of exogenous agents, bioeffects are generated due to a direct interaction between the ultrasound pressure wave and tissue. The precise bioeffect depends on exposure conditions, with modulation of gene expression and increased perfusion for mild acoustic outputs, and ablation associated with higher acoustic outputs.^33^, ^34^ A wide range of ultrasound‐induced mechanisms are responsible for generating these bioeffects, including heating, streaming, radiation force, and cavitation generated de novo (Figure 2).

### Low‐Intensity Pulsed Ultrasound

Ultrasound exposure within and just beyond diagnostic acoustic output levels have been investigated for therapeutic benefit (1–3 MHz, 0.02–1 W/cm^2^).^35^ Low‐intensity pulsed ultrasound (LIPUS) has been categorized as insufficient to generate significant thermal effects, but can cause tissue displacement or fluid streaming due to radiation force.^36^ Given the low acoustic output of LIPUS systems, cavitation effects are unlikely without the addition of exogenous agents.^31^

#### Bone Fractures

The application of LIPUS to aid in bone fracture healing was approved by the U.S. FDA in 1994,^37^ and has been shown to be effective at each stage of tissue remodeling.^38^ Several ultrasound‐induced bioeffects may contribute to the healing process,^39^, ^40^, ^41^, ^42^ though the variation in exposure conditions between studies limits identifying a cohesive narrative. Nevertheless, bone healing remains the most studied area of LIPUS, and is the only FDA‐approved indication.

#### Neuromodulation

Transcranial LIPUS has been shown to promise in altering neural circuits in the brain.^43^ Specifically, low‐frequency ultrasound interacts with calcium‐selective mechanosensitive ion channels.^44^ The resulting outcome is modulation of the long‐term plasticity and short‐term excitability and connectivity in the brain.^45^, ^46^, ^47^ Studies in non‐human primates demonstrate that LIPUS can achieve sustained effects in deep‐brain circuits and oculomotor performance.^48^, ^49^ Single‐neuron discharge in macaques has been recorded during ultrasound exposure, providing direct evidence of neuromodulatory effects.^50^ Another interesting outcome of LIPUS‐induced neuromodulation has been the induction of torpor in rodents, resulting in a hypothermic and hypometabolic state.^51^ This finding provides insights to the hibernation behavior of mammals and has applications for long‐term space travel and slowing disease progression.

Neuromodulation has been explored in a range of conditions, including chronic pain, disorder of consciousness, Alzheimer's disease, Parkinson's disease, depression, schizophrenia, anxiety disorders, substance use disorder, drug‐resistant epilepsy, recovery from stroke, dementia, and traumatic brain injury.^45^, ^52^ No safety concerns were noted in these studies, suggesting neuromodulation with LIPUS may be considered as a therapeutic tool and for scientific investigation.^53^ A working group has been formed to develop standardized protocols to report exposure parameters for LIPUS neuromodulation studies. A goal of these protocols is to facilitate the use of neuromodulation in research and clinical translation.^54^ Indeed, several clinical trials are ongoing to explore potential applications (NCT06249711, NCT06453109).

#### Sonogenetics

The goal of sonogenetics is to modulate cell activity through engineered or modified protein mediators that confer or amplify sensitivity to ultrasound.^55^ Early sonogenetic studies focused on ultrasound‐induced gene switch activation.^56^, ^57^ Its current focus is geared toward findings in the neuromodulation community.^58^ Other potential applications include diseases of the central and peripheral nervous system,^59^ retinal cells,^60^ and stimulation of neurons for vision restoration.^61^

Mechanically driven sonogenetic effects were first demonstrated in nematodes (*Caenorhabditis elegans*) modified to express the stretch‐sensitive mechanotransduction cation channels TRP‐4 and MEC‐4. The locomotional behavior of modified nematodes was altered following exposure to LIPUS.^62^ These results were promising, although TRP‐4 and MEC‐4 do not translate into mammals. Applicable genes have been identified that can be modified that express mechano‐ or thermo‐sensitive proteins.^63^, ^64^ Current approaches focus on altering the intercellular calcium concentration.^44^, ^65^ Ultrasound‐induced hyperthermia (see Mild Hyperthermia section) in combination with temperature‐actuated switches has been investigated for activation of immune checkpoint inhibitors in engineered bacteria.^66^ This approach has also been shown to regulate chimeric antigen receptor T cells (CAR‐T cells) in tumor environments.^67^

### Mild Hyperthermia

Hyperthermia refers to the heating of tissue to temperatures in the range of 41–45°C for durations of ~30–60 minutes. These effects are achieved with acoustic powers of approximately 10–100 W.^68^, ^69^, ^70^ Mild heating has been shown to combine synergistically with other cancer therapies.^71^, ^72^, ^73^ Hyperthermia has been shown to enhance tissue damage from radiation therapy,^74^, ^75^ and including in clinical trials for multiple tumor types.^73^ Other applications of hyperthermia include enhanced drug delivery using thermosensitive liposomes^76^, ^77^, ^78^ and enhanced immune effects for CAR‐t cell immunotherapy.^79^ The systems used to produce acoustic fields for hyperthermia include single or multi‐element sources, phased arrays, or intracavity devices.^72^, ^80^ Volumetric heating can be accomplished through mechanical scanning, and/or phased arrays that offer electronic beam forming and steering. Recent investigations demonstrated the feasibility to design holographic lens transducers that produce uniform thermal dose profiles over arbitrary tumor volumes.^81^

### Thermal Ablation

The application of focused ultrasound to cause thermal ablation has been extensively investigated for multiple targets. Focused ultrasound was applied to more than 98 k patients worldwide in 2022, the majority of which were thermal ablation cases.^82^ The typical fundamental frequency for thermal ablation ranges from 0.5 and 10 MHz, predominantly at the lower end of this spectrum.^83^ Focused pulses have spatial‐peak, time‐average intensities (*I*_*SPTA*_) from 0.001 to more than 1 kW/cm^2^. The resultant effect is the generation of in situ temperatures ranging from 48°C to more than 70°C that causes cell death in minutes to seconds, respectively.^84^ Therapeutic volumes larger than the focal region are achieved by targeting multiple locations sequentially or in an interleaved fashion.^85^ For all but the most superficial targets, image guidance is employed with ultrasound or magnetic resonance imaging (MRI).

Many applications of thermal ablation have transitioned into clinical use since 2012. The Insightec Exablate Neuro received U.S. FDA approval for unilateral thalamotomy to treat essential tremor in 2016. The indications for use were expanded to include tremor‐dominant Parkinson's disease in 2018.^86^, ^87^ A thermal lesion generated within the ventral intermediate nucleus ablates misfiring neurons responsible for symptoms.^88^ This system uses a hemispherical array of 1024 elements to deliver transcranial ultrasound corrected for phase aberrations caused by the skull. The corrections are determined through simulations based on computed tomography scans of the skull. In situ corrections are also applied based on MRI thermometry to track mild heating from sub‐therapeutic test pulses. Thalamotomy to treat tremor syndromes targets volumes of 100–200 mm^3^ at maximum temperatures around 56°C.^89^ A large clinical trial demonstrated significant improvement in hand tremor scores with persistence of 36 months.^90^ The Exablate Neuro is in clinical trials underway for targeting pediatric tumors (NCT03028246) and gliomas (NCT03100474). A trial targeting other brain tumors with the Exablate Neuro system was recently completed (NCT01698437).

The Sonalleve system was designed to target uterine fibroids, and has been adapted to treat primary and secondary bone cancers.^91^, ^92^ Thermal ablation has also been approved for palliative care of bone metastases with the Sonalleve.^93^ Focused ultrasound is applied to disrupt nerves endings on the periosteum of the diseased bone to alleviate pain, not the lesion itself.^94^ Thermal ablation has been cleared for other benign musculoskeletal tumors, such as osteoid osteomas.^95^ For patients with hypertension, thermal ablation is being applied to tissue surrounding the main renal arterial supply.^96^, ^97^ The goal of this trial is to decrease the over‐activity of nerves and reduce blood pressure (NCT02649426). Multiple focused and unfocused systems have been developed and approved for prostate ablation.^98^, ^99^, ^100^, ^101^, ^102^ A healthcare cost analysis indicated focused ultrasound had lower expenditures than radical prostatectomy and external beam radiotherapy as primary treatment options.^103^ There is also ongoing work to treat liver,^85^ kidney,^85^ breast cancers,^104^ thyroid nodules,^105^ desmoid tumors,^106^ and nerves^107^ with thermal ablation.

### Catheter‐Based Ultrasound

Intravascular catheter devices are used commonly by physicians to perform minimally invasive procedures. Clinical studies have demonstrated the safety and efficacy of the EKOS system, an FDA‐cleared ultrasound‐assisted, catheter‐directed thrombolysis device to treat pulmonary embolism.^108^, ^109^, ^110^ The device emits cylindrically symmetric ultrasound pulses within the thrombus between 2.0 and 2.25 MHz fundamental frequency, 1.5 MPa peak negative pressure, and 15% duty cycle.^111^ Thrombolytic therapy is administered through infusion ports in the catheter during insonation. A clinical study regarding the benefit of the EKOS system was inconclusive, potentially due to insufficient statistical power in the experimental design.^112^ An ongoing, multinational/multicenter/randomized/controlled clinical trial is underway designed to provide sufficient data to compare EKOS with an anticoagulant with anticoagulant alone.^113^ In addition to pulmonary embolism, pre‐clinical studies have indicated that the EKOS system provides effective drug delivery for the treatment of peri‐stent restenosis.^114^, ^115^.

Other catheter‐based ultrasound systems have been developed to treat vascular calcifications. Severe vascular calcification can inhibit balloon expansion during angioplasty and stent placement in a stenotic vessel.^116^ An FDA‐approved device has been developed for intravascular lithotripsy to disrupt calcifications mechanically.^117^, ^118^, ^119^, ^120^, ^121^ An electric spark discharge produces ultrasound waves that interact with calcifications in an analogous fashion to lithotripsy for renal calcifications (see Kidney Stone Management section).^122^ The system has been applied to peripheral and coronary targets, each of which requires a different number of pulses for effective treatment.

### Histotripsy

Mechanical bioeffects can also be used to ablate tissue. Histotripsy (*histo*: cells; *tripsy*: breaking) applies pulses 1 μs to 10 ms in duration with much higher spatial‐peak, pulse‐average intensities than used in thermal ablation to generate inertial cavitation (> 20 kW/cm^2^ for histotripsy compared to 0.001 kW/cm^2^ for thermal ablation).^123^, ^124^ Bubbles formed in the focal zone fractionate cells without heating the target.^125^ There are multiple types of histotripsy, each of which use different mechanisms to generate bubbles.^126^, ^127^ Intrinsic‐threshold histotripsy applies pulses of 1 cycle with a peak negative pressure that exceeds ~25 MPa.^31^ Bubbles are generated due to the tension of the ultrasound pulse. Shock‐scattering histotripsy uses highly nonlinear pulses 3 to 20 cycles in duration, with peak negative pressures of 15 MPa or greater. A cloud of bubbles is formed due to interactions between the incident pulse, and the shock wave of the pulse that scatters from bubbles formed in the focal zone.^128^ Boiling histotripsy relies on pulses 1–10 ms in duration that cause rapid shock wave‐induced heating.^129^ The increased temperature lowers the peak negative pressure required to cause bubble nucleation.^130^ To date (2024), clinical trials are underway or have been completed to test the safety and technical success of histotripsy technology for the treatment of prostate tissue (NCT01896973), liver (NCT04572633), kidney (NCT05820087), pancreatic adenocarcinoma (NCT06282809), and calcified aortic stenosis (NCT03779620). Further, the FDA granted a de novo request for this technology in the treatment of lesions in the liver.

Pre‐clinical studies have demonstrated that histotripsy sensitizes pathologies to other therapeutic approaches. Bubble activity decellularizes tissue, but it is not as effective for breaking down stiff extracellular structures.^131^ This property of histotripsy can be advantageous for targets that encompass major vessels with extensive collagen and fibrin.^132^ Extracellular structure can be problematic for applications like venous thrombosis, where fibrin will be undertreated and may serve as a nidus for re‐thrombosis.^133^ Combining histotripsy with a fibrinolytic drug has been shown to treat the cellular and extracellular clot components synergistically.^134^ Histotripsy has been also shown to enhance the delivery of doxorubicin in a murine model of pancreatic cancer.^135^

Systemic bioeffects have been observed when histotripsy is applied to free‐flowing blood or venous thrombosis without sufficient anticoagulation. The mortality rate of swine in these studies was 45–50% compared to 0% when heparin was administered during insonation.^136^, ^137^ The precise cause of mortality in these studies is unknown. There were no cases of vascular perforation or pulmonary embolism. Histotripsy causes significant hemolysis,^134^ which is a pathway for platelet activation and intravascular thrombosis.^138^ Microclotting was observed in the heart and lung of pigs that expired during treatment,^137^ consistent with platelet‐induced intravascular thrombosis. Spontaneous thrombus formation may be beneficial when targeting certain lesions. Histotripsy has been applied successfully across the capsule of the liver and kidney without extraneous bleeding issues. This was accomplished when therapeutic and supratherapeutic dose of the antithrombotic warfarin were administered.^139^ The lack of excessive bleeding was attributed in part due to thromboses in vessels that coincided with the treatment zone. The thrombus resolved over time, as evidenced by patent vessels in follow‐up contrast‐enhanced imaging.^140^ Note that the antithrombotic warfarin is not an anti‐platelet agent.^138^

### Kidney Stone Management

#### Shock Wave Lithotripsy

Among the primary uses of therapeutic ultrasound, shock wave lithotripsy has been part of the standard‐of‐care for kidney stones since the 1980s.^141^ The primary goal of lithotripsy is to break down mineralized structures to return the patient to homeostasis. As a result of lithotripsy treatment, kidney stones are reduced to a manageable size that can be passed naturally. Shock wave lithotripsy is also used to target gallstones.^142^ Lithotripters are often focused sources that generate shock waves within the treatment zone. The first sources were electrohydraulic, using a spark gap at one focus of a truncated ellipsoidal reflector to generate a shock wave. Subsequent revisions to lithotripsy devices rely on electromagnetic and piezoelectric sources. Multiple mechanisms are responsible for stone erosion, including inertial cavitation, spallation (reverberations of ultrasound waves within the mineralization), shear stresses, super focusing, squeezing, and fatigue.^143^

Though shock wave lithotripsy remains the primary intervention for certain stone types, its use has declined in recent years in favor of ureteroscopy devices.^144^, ^145^ In a recent meta‐analysis, ureteroscopy devices were found to have more favorable outcomes than shock wave lithotripsy for stones 1–2 cm in diameter in terms of total removal, retreatment, and need for auxiliary approaches.^146^ For the same group of stones, shock wave lithotripsy was preferable in terms of the procedure duration and time to recovery. No differences were noted between the therapies for stones less than 1 cm diameter. Shock wave lithotripsy retains advantages over ureteroscopy, including a reduced impact on patient quality of life, and shorter hospital stays.

#### Burst Wave Lithotripsy

Spallation and shear stresses are among the mechanisms of kidney stone degradation under shock wave lithotripsy.^147^ Burst wave lithotripsy accentuates these mechanisms through applying tone bursts of 100–800 kHz that are lower in amplitude relative to shock wave lithotripsy (a shock amplitude of 30–100 MPa for shock wave lithotripsy compared with peak pressures of 2–6 MPa for burst wave lithotripsy).^148^, ^149^ Stones treated with burst wave lithotripsy acquire periodic fracturing throughout the structures prior to fragmentation. The periodicity of fractionation is proportional to the wavelength of the tone burst, providing a means to “dial in” the size of residual debris. In contrast, shock wave lithotripsy tends to bisect the stone. These outcomes appear to be due to differences in scattering within the stone based on data collected in a photoelastic model stone that was used to visualize stress waves.^150^ Shock wave lithotripsy produced a single region of high tensile stress via spallation, whereas agrid of standing waves were produced in burst wave lithotripsy.

There is also a fundamental difference in bubble activity between the 2 lithotripsy approaches. Cavitation can be a prominent feature of shock wave lithotripsy due to the long‐duration peak negative phase of the pulse combined with the shock wave.^149^ The nearly linear excitations for burst wave lithotripsy with relatively low pulse peak negative pressures minimize bubble activity.^148^ These features allow burst wave pulses to be delivered at a considerably higher rate (10–100 Hz) compared to shock wave lithotripsy (1–2 Hz) without producing cavitation in vivo.^151^ Further, pre‐clinical studies have demonstrated that burst wave lithotripsy does not cause hemorrhagic injury or alter renal function.^152^ This finding is particularly important given other extracorporeal stone removal procedures are associated with more than a 50% decline in renal function after treatment.^153^

The technology was tested in a prospective, multi‐institutional feasibility study (NCT03873259).^154^ Subjects screened for at least 1 stone less than 12 mm in diameter via computerized tomography were recruited for testing. The primary outcome was safety. Injury was found to be negligible and included mild reddening of the papilla with some hematuria. In terms of efficacy, 91% of stones showed fragmentation. Of these, 39% were fragmented completely within 10 minutes. These results indicate burst wave lithotripsy results in comminution of the total stone volume into fragments for 90% of cases with only mild tissue injury.

#### Intracorporeal Lithotripsy

Minimally invasive intracorporeal devices are also available to achieve stone comminution. Guidelines from the American Urological Association and the Endourology Society recommend percutaneous nephrolithotomy as the first line of therapy for symptomatic patients with a total kidney stone burden greater than 20 mm.^155^ This system utilizes a rigid endoscope to place a comminution device near the stone. Current devices rely on energy generated by ultrasound, pneumatic, or laser sources, or a combination thereof. Ultrasound‐based devices use piezoelectric elements with a center frequency of ~24 kHz or an electrohydraulic spark discharge to create the pressure waves, and potentially cavitation, responsible for stone comminution.^156^ Similar stone clearance rates were found in a prospective study comparing ultrasound and pneumatic devices.^157^ A retrospective study found that an ultrasound systems performed the same or better than a laser‐based device, though was dependent on stone hardness.^158^

#### Ultrasonic Propulsion

Following the fragmentation of kidney stones, clearance of residual debris is among the primary considerations in the effective management of the disease.^159^ Passage of fragments can be affected by several factors, including infundibulopelvic angle, infundibular length, and spatial orientation of caliceal anatomy.^160^ Acoustic radiation force is one means to address fragments lodged in the lower pole.^19^ Early studies demonstrated the feasibility to displace kidney stones at velocities up to 1 cm/s with radiation force generated by focused ultrasound.^161^ Refinement of the technology has advanced to vortex beams that use acoustic trapping to displace the stone.^162^ Here, the vortex is created by varying the phase of the wave emitted across the transducer surface to generate a helical wavefront. The outcome is an intensity ring in the plane orthogonal to the beam axis. The force of the high‐intensity ring pushes an object (such as a kidney stone) toward the center of the ring. Prototypes for this vortex beam manipulated model kidney stones of millimeter size in 3 dimensions both in vitro and in the kidney of a pig. Deviations of the stone motion remained on average within 10% of the intended path, with no injury to the bladder wall or intervening tissue.

First‐in‐human trials of ultrasonic propulsion relied on modified C5‐2 curvilinear probe (Philips Ultrasound, Andover, MA) driven by a research platform (VAS, Verasonics Inc, Redmond, WA),^163^ including a low output setting for stones at depths less than 7 cm, and a high output setting for stones at greater depths. A maximum of 40 pushes were applied to each patient. An initial 15‐patient trial demonstrated movement in 65% of targets, with 30% moving more than 3 mm. The maximum stone displacement was 10 mm. Over 30 fragments were passed in 4 of 6 subjects that had previously undergone a lithotripsy procedure. Discomfort during the procedure was rare, mild, brief, and self‐limited. An updated device was tested in a follow‐up single‐institution trial (NCT02028559). This device updated the C5‐2 system by increasing the pulse duration (2–5 seconds, 5–40 W), a limiting factor to the amount of radiation force applied to the stone. Further, the new instrumentation improved the penetration depth for the pushing pulse and had a broader beamwidth to provide better manipulation of stone fragments.^164^ Successful movement of at least 1 stone target was achieved in 95% of cases, with no notable influence of body mass index or other features.^165^ No serious or unanticipated adverse events related to the treatment were noted, consistent with prior clinical and pre‐clinical studies.^165^

### Physical Therapy

Ultrasound can be used to alleviate injuries that limit mobility or the performance of daily functions, such as osteoarthritis, soft tissue injuries, and myofascial pain. Unfocused ultrasound sources with low spatial‐average, temporal‐average intensities (0.02–2.5 W/cm^2^), heat‐injured tissue to increase blood flow, and oxygen delivery to accelerate healing.^166^, ^167^ However, there is debate in the physical therapy community regarding the benefits of heating for these injuries.^168^

Extracorporeal shock waves have been used to address musculoskeletal disorders such as plantar fasciitis and lateral epicondylitis. Success rates for shock wave therapy range from 30 to 90% in muscular applications (eg, plantar fasciitis, lateral epicondylitis, calcified tendinitis of the shoulder, and Achilles tendinopathy) and 50–85% in bone disorders (eg, non‐union and delayed union of long bone fracture).^169^ The therapeutic mechanisms of shock waves for benefit in physical therapy are not fully understood. The primary hypothesis is that shock waves cause interstitial and extracellular responses that promote tissue regeneration.^170^ In addition to these direct effects of the shock wave, cavitation generated via boiling histotripsy has been investigated to address Achilles tendinopathy as an alternative to dry needling.^171^ Purely mechanical disruption was achieved with pulse durations of 0.1–1 ms in the form of fiber separation and fraying. A combination of thermal and mechanical damage was observed for longer pulse duration, indicating that a range of exposure parameters can be identified that achieve tendon disruption.

### Cosmetic Ultrasound

Esthetic medicine focuses on improving the appearance of patients. Therapeutic claims described for ultrasound in cosmetic medicine include fat reduction,^172^ wrinkle smoothing,^173^ tightening of loose skin areas,^174^ stretch mark removal,^175^ cellulite/orange‐peel skin treatment,^176^ spider veins, telangiectasis,^177^ abnormal pigmentations, acne, and scar reduction.^178^ Reported studies testing ultrasound for cosmetic purposes have often been industry sponsored. There have been no randomized controlled trials, although a few clinical studies have compared treated and untreated sites within subjects.^179^ Most reports have been based on single‐center studies ranging in size from 6 to 152 subjects. Use of analgesia with paracetamol, or of anesthesia, has been sporadically reported. Reported adverse effects range from mild transient pain, erythema and edema of skin lasting a few days,^180^ to more prevalent and wide‐ranging effects,^181^ including pain after treatment (75%), edema (75%), bruising (66%), pain during treatment (66%), tingling (60%), erythema (45%), and skin burns (2/152 = 1.3%) that can occur at the second‐degree level (1/152 = 0.65%). Pain scores were higher when higher energy levels were used.^182^ In several studies where blood lipids and other liver function tests were examined, no anomalies were detected after treatment.^183^, ^184^ Some studies have reported the appearance of hard subcutaneous nodules, a burning sensation, mild blisters, and 1 case of purpuric lesions.^183^, ^185^, ^186^ In general, patients describe most adverse effects resolved spontaneously within 4–12 weeks.

### Immnuno‐oncology

The immunostimulatory effects induced by ultrasound also hold promise in cancer treatment.^187^, ^188^, ^189^, ^190^ There are 2 distinct translational strategies. The first strategy targets diseases with a poor response to existing immunotherapies (eg, breast, ovary, prostate, pancreas, and primary brain tumors). The goal of this approach is to initiate or modulate an anti‐tumor immune response. The second strategy seeks to boost treatment efficacy or delivery in cancers responsive to immunotherapies (eg, melanoma, kidney, and liver).

Histotripsy, thermal ablation, and hyperthermia have been explored for their immunological impacts.^79^ Approaches that use exogenous microbubble nucleation agents in combination with ultrasound have also been shown to promote an immune response.^191^ Mechanical ablation with histotripsy can initiate damage‐associated molecular patterns (DAMP) release, modulate cytokine and chemokine profiles, and reduce pro‐tumor immune cells.^192^ Increased CD8+ infiltration was found in murine subcutaneous melanoma tumor (B16GP33 cell line) following histotripsy relative to radiation therapy or radiofrequency ablation.^193^ These outcomes were hypothesized to result from preservation of tumor‐presenting antigens under histotripsy, in contrast to thermal ablation which can denature protein structures.^194^ Changes in tumor oxygenation due to histotripsy may also contribute to the observed immune response. Assessment of neuroblastoma tumor following histotripsy exposure in a murine orthotopic model indicated mitigation of tumor hypoxia, a primary prognostic of disease resistance.^195^ These shifts were attributed to the proliferation of vasculature surrounding the treatment zone, providing means for blood flow and reoxygenation in the targeted tumor.^196^ Thermal treatments alter vascular permeability/perfusion, induce heat shock proteins and pro‐inflammatory cytokines/chemokines, boost immune cell infiltration and the cytotoxic activity of natural killer and CD8+ T cells.^79^ Partial ablation strategies during thermal ablation might further enhance immune cell infiltration.^197^ These effects can vary with tumor type. For instance, fibrous tumors like pancreatic cancer may benefit from mechanical disruption of the extracellular matrix to facilitate immune cell penetration infiltration.^198^ Focused ultrasound and microbubbles have been used for blood–brain barrier (BBB) opening, which induce inflammation to neurogenesis stimulation, and modulation of microglial structure.^199^, ^200^, ^201^, ^202^ Clinical studies have documented immuno‐modulation of cancers, including liver,^203^ breast,^204^, ^205^ thyroid,^206^ and others.^207^ Notably, thermal ablation of breast cancer results in an increase of activated dendritic cells, macrophages, and B lymphocytes.^205^, ^208^ Combining sonodynamic therapy (see Sonodynamic Therapy section) with anti‐PD1/PD‐L1 therapies initiates a favorable adaptive immune response in multiple tumor models.^209^ More immuno‐competent tumors with pre‐existing anti‐tumor immune cells may respond better to non‐ablative, lower‐power treatments that amplify cytokine signaling without harming infiltrative immune cells.^210^ Some tumors with significant pro‐tumorigenic infiltration might require more aggressive ablative methods.^204^

Control of local diseases and at distant sites (abscopal effect) has been reported in case reports for thermal ablation of pancreatic cancer^211^ and mechanical ablation of liver cancer.^212^ There is a lack of evidence that therapeutic ultrasound alone can have an immuno‐stimulatory effect strong enough to be curative. Several combinatorial approaches have been tested in preclinical tumor models, including thermal ablation with toll‐like receptor agonist CpG and anti‐PD1 in breast tumor models.^213^, ^214^ Histotripsy has been tested in combination with anti‐CTLA‐4 and anti‐PD‐L1 for neuroblastoma,^215^ with anti‐PD1 and CAR T cells for brain tumors,^216^ and anti‐CTLA‐4 or CD40 agonist for melanoma.^193^, ^217^ Clinical trials are ongoing to assess the potential of combination treatment, including thermal ablation with checkpoint inhibitors to treat breast cancer and solid tumors (NCT04116320), and ultrasound and microbubble BBB opening to deliver nivolumab in melanoma brain metastases (NCT04021420) or pembrolizumab for recurrent glioblastoma (NCT05879120). Partial thermal ablation of tumors is also being tested to promote immune responses for undifferentiated pleomorphic sarcoma (NCT04123535). Key unresolved issues include determining effective exposures to generate the intended bioeffect, the sequence for combined therapies, and methods to monitor the immunological impacts.

### Skin Permeabilization

The skin presents a large and easily accessible pathway for the delivery of therapeutics to the body. Drugs that are hydrophilic or greater than 500 Daltons have limited penetration across the skin,^218^, ^219^ and require injection or surgical approaches for administration. Ultrasound has been shown to permeabilize the skin in both animal models and human skin,^220^ and for a wide variety of drugs ranging from molecules to microparticles.^221^ Both thermal and cavitation effects have been indicated as a possible mechanism of action. Ultrasound frequencies from 100 kHz to greater than 10 MHz have been investigated, and may contribute to variability of cavitation from endogenous nuclei.^222^, ^223^ Exogenous microbubble nucleation agents are also employed to achieve the intended bioeffects (see Types of Exogenous Agents section).^224^ Ultrasound can be administered through either traditional piezoelectric transducers or wearable devices.^225^

## Ultrasound Therapy with Exogenous Agents

**Figure 3:**
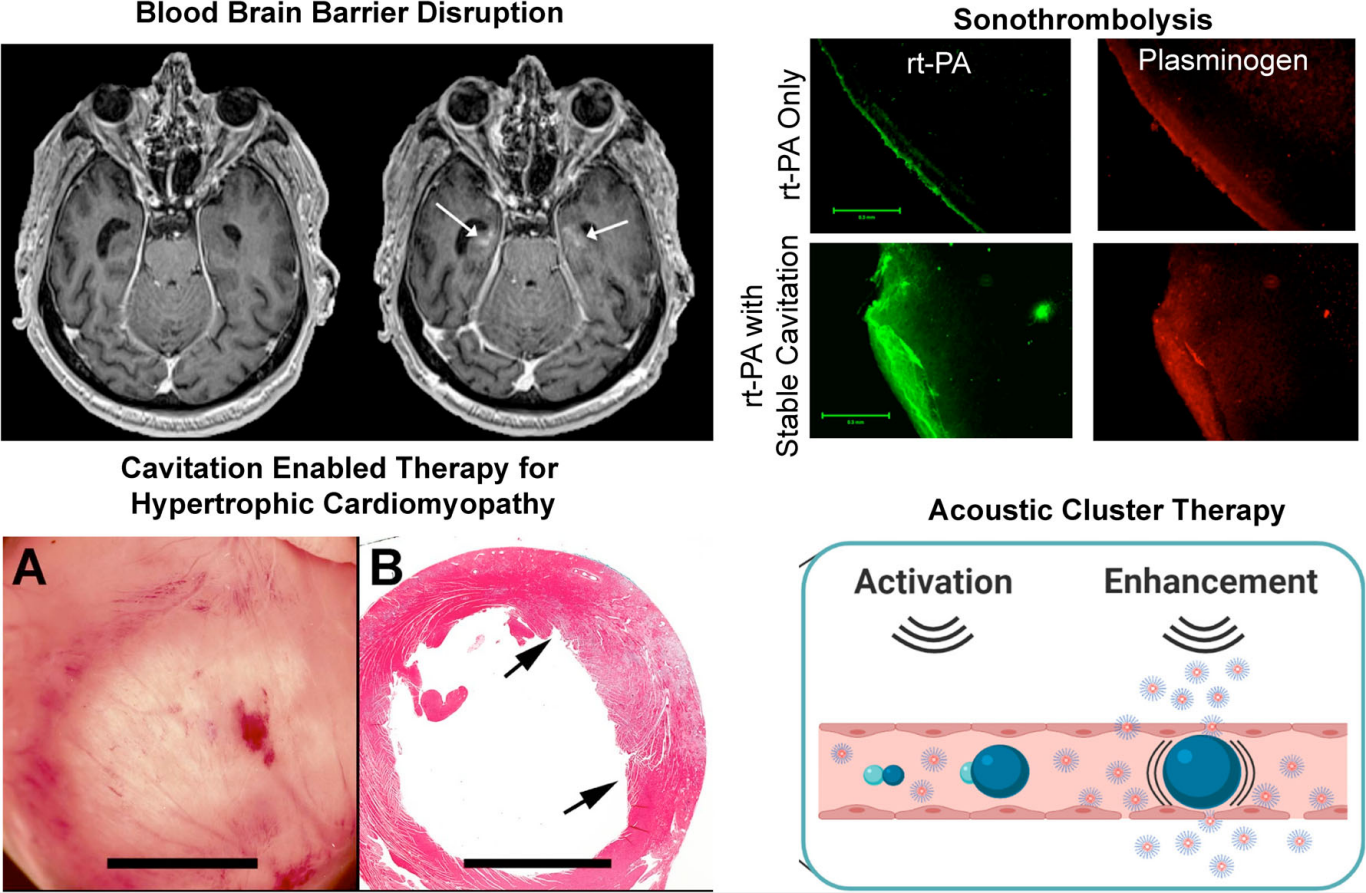
A subset of therapeutic ultrasound applications that rely on exogenous agents to generate bubble activity. *Blood–Brain Barrier Disruption* uses transcranial or intracranial ultrasound in combination with microbubbles to enable large molecule drugs to pass from the vasculature into brain matter. An increase in brain permeability is apparent pre (left) and post (right) via contrast extravasation on T1‐weighted MRI (arrows, Reprinted from *Brain*, vol. 146, no. 3, Blood–brain barrier opening of the default mode network in Alzheimer's disease with magnetic resonance‐guided focused ultrasound, pp. 865–872, Copyright 2023, by permission of Oxford University Press). *Sonothrombolysis* enhances the penetration of a thrombolytic drug (eg, recombinant tissue plasminogen activator, rt‐PA) via microbubble activity into thrombus (left column) to promote fibrinolysis as indicated by plasminogen, a step in the fibrinolysis process highlighted in right column (Reprinted from *Ultrasound in Medicine and Biology*, vol. 34, no. 9, Ultrasound‐enhanced thrombolysis using Definity as a cavitation nucleation agent, pp. 1421–1433, Copyright 2008, by permission of Elsevier under creative commons license CC BY‐NC‐ND 4.0). *Cavitation‐enabled therapy for hypertropic cardiomyopathy* generates microlesions to reduce excessive heart muscle bulk. The treated region (light area in left image) indicated inflammation 2 days after bubble activity concurrent with swelling (arrows in right image) and inflammatory cells indicated via blue‐stained nuclei (Reprinted from *Ultrasound in Medicine and Biology*, vol. 44, no. 7, Ultrasound cavitation‐enabled treatment for therapy of hypertrophic cardiomyopathy: Proof of principle, pp. 1439–1450, Copyright 2018, by permission of Elsevier under creative commons license CC BY‐NC‐ND 4.0). *Acoustic cluster therapy* relies on a microbubble/droplet moiety to embolize vasculature to increase the penetration of therapeutics into the target (Reprinted from *Journal of Controlled Release*, vol. 10, no. 337, Acoustic Cluster Therapy (ACT®) enhances accumulation of polymeric micelles in the murine brain, pp. 285–295, Copyright 2021 under creative commons license CC BY 4.0).

Cavitation is among the most common mechanism used for therapeutic ultrasound. Endogenous bubble nuclei must be activated by specialized driving electronics to generate reliable activity.^31^ Exogenous nucleation agents lower the peak negative pressures required to generate cavitation,^226^, ^227^, ^228^ thereby expanding the number of targets that can be treated safely with mechanical bioeffects (Figure 3).

### Types of Exogenous Agents

#### Ultrasound Contrast Agents

There are several commercially available ultrasound contrast agents that have been approved by the FDA for diagnostic imaging. Ultrasound contrast agents are the most widely investigated cavitation nuclei in investigated in therapeutic ultrasound, as reported previously.^32^, ^229^ Lipid shells counter the effects of surface tension to increase the persistence of ultrasound contrast agents in whole blood,^230^ though other stabilizing materials have also been investigated.^231^ A gas with low solubility in blood relative to air (eg, sulfur hexafluoride or perfluorocarbon) is used to increase the half‐life of the contrast agent.^232^, ^233^ Bioactive gasses that release upon rupture of the contrast agent have also been explored.^234^, ^235^ Approved agents are typically between 1 and 10 μm in diameter,^236^ though advanced formulation methods have been developed with microfluidics to produce size‐isolated distributions.^237^, ^238^, ^239^, ^240^ Multi‐functional vesicles have been developed that incorporate drug‐loading onto the microbubble shell,^230^ or integrate the microbubble into a multilamellar structure that encapsulates the therapeutic.^241^ Targeting ligands have also been incorporated into the shell to localize therapeutic effects to a specific biomarker.^32^

#### Ultrasound‐Activated Droplets

Liquid droplet agents have been explored for therapeutic applications as an alternative to microbubbles. Droplets are commonly formed with a liquid perfluorocarbon core with a transition temperature near physiologic conditions. The perfluorocarbon liquid is in a metastable state, and transitions to a gas microbubble following ultrasound exposure.^242^, ^243^, ^244^, ^245^, ^246^ This method of creating microbubbles in situ is known as acoustic droplet vaporization, and has been found to be well tolerated in vivo.^247^ A shell comprised of polymers, proteins, or lipids is used to stabilize the aqueous phase.^245^, ^248^, ^249^ There are numerous manufacturing methods for droplets, including amalgamation, condensation, sonication, extrusion, high‐shear homogenization, and microfluidics. Each process generates different ranges of droplet size (100 nm to more than 10 μm) and polydispersity.^242^, ^250^, ^251^, ^252^, ^253^, ^254^, ^255^, ^256^ Multiple studies have been published to understand the physical processes that lead to vaporization,^246^, ^257^ including the exposure parameters required for droplet transition, and the portion of the droplet population that transitions.^258^, ^259^, ^260^, ^261^, ^262^

Initial studies in therapeutic applications of droplets focused on embolotherapy to reduce perfusion that causes detrimental cooling during thermal ablation.^263^ Droplet vaporization embolization has been applied to increase drug uptake into targeted tissues.^264^, ^265^, ^266^ Droplets have been tethered with microbubbles to reduce the pressure required for embolization (ie, acoustic cluster therapy), and are undergoing testing in a clinical trial (NCT04021277). Drug delivery has been further enhanced by co‐administering droplets and a pharmacologic therapeutic,^267^, ^268^ or binding the pharmacologic therapeutic to the droplet surface.^269^, ^270^, ^271^ Double emulsions have also been developed for volumetric therapeutic loading.^260^, ^272^, ^273^, ^274^ The in situ formation of microbubbles has been demonstrated to accelerate ablation with focused ultrasound through thermal^275^, ^276^, ^277^, ^278^ and mechanical^279^, ^280^, ^281^, ^282^, ^283^ approaches. The unique ability of perfluorocarbons to solubilize oxygen has also led to pre‐clinical studies to modified dissolved gas concentrations.^284^, ^285^

#### Other Agents

Additional agents include nanobubbles,^286^ and gas‐stabilizing solid cavitation agents.^287^ Through an interfacial seed polymerization method,^288^ nanocups have been manufactured from polystyrene spheres.^289^ The hydrophobic surface of the nanocup harbors a gaseous core that undergoes inertial cavitation at peak negative pressures between 0.5 and 1 MPa.^290^ Due to their relatively small size compared to microbubbles (100–500 nm versus 1–10 μm), nanocups, nanodroplets, and nanobubbles can efficiently penetrate leaky tumor vasculature.^32^, ^291^ Other potential applications for nanocups include transdermal transport of vaccines and delivery of oncolytic vaccinia viruses.^292^, ^293^ Similar nanocone formulations have been investigated for histotripsy.^294^

Another form of nucleation agent under development is gas vesicles, a genetically encodable, air‐filled protein nanostructure. These nanostructures evolved in photosynthesis bacteria and archaea to achieve cellular buoyancy.^295^ Multigene clusters within bacteria and mammalian cells can be modified to encode gas vesicles that can be used as ultrasound contrast agent activated via gene expression.^296^, ^297^, ^298^ Gas vesicles hone to specific cell types, which make them ideal for targeted ablation therapies. The capacity of gas vesicles to generate both stable and inertial cavitation under the action of focused ultrasound has been validated in vitro.^299^ Based on these findings, gas vesicles have been generated for treating cancer through modifying tumor‐homing bacteria. Outcomes were tested in a murine subcutaneous tumor treated in parallel with the administration of checkpoint inhibitors known to be synergistic with mechanical tumor disruption. The combined effects of gas vesicles, focused ultrasound, and checkpoint inhibitors increased survival by a factor of 2 relative to the checkpoint inhibitor alone. In addition to ablation, gas vesicles have been proposed to enhance radiation force on cells for applications such as tissue engineering.^300^

### Blood–Brain/Blood–Spinal Cord Barrier

First demonstrated in 2001,^301^ focused ultrasound insonation of circulating microbubbles is capable of safely and reversibly increasing the permeability of the BBB and blood–spinal cord barrier (BSCB) using stable cavitation.^302^, ^303^ The BBB and BSCB exclude most drugs from extravasating into the brain or spinal cord, which complicates treatment for malignancies in these biological structures. Ultrasound‐mediated BBB and BSCB disruption has enabled the delivery of multiple drugs, antibodies, genes, viruses, and even cells in pre‐clinical animal models.^304^, ^305^, ^306^

Ultrasound‐mediated BBB disruption was first tested clinically to treat Alzheimer's disease in 2018.^307^ In this clinical trial, the primary goal was technical success of BBB disruption. The primary safety endpoints were met, which demonstrated the BBB could be reversibly disrupted without hemorrhage, swelling, or neurological deficits. No clinically significant change in patient cognition or function was observed. A pre‐clinical study indicated BBB disruption alone is capable of reducing amyloid‐β plaque burden in a mouse model of Alzheimer's disease.^308^ Reduction in amyloid‐β has also been observed in patients for BBB opening only,^309^, ^310^, ^311^, ^312^ and when focused ultrasound was combined with aducanumab.^313^ Imaging with 18F‐florbetaben PET indicated the amyloid‐β plaque burden was reduced in regions targeted with focused ultrasound relative to the control hemisphere after 26 weeks.

Several other clinical BBB opening studies are ongoing, including the delivery of chemotherapy for Her2‐positive brain metastases (NCT03714243). Another trial is ongoing that seeks to deliver the synthetic enzyme Cerezyme to the brains of patients with Parkinson's disease (NCT04370665). A phase 1 trial is underway to test the feasibility of BBB opening to enhance the delivery of paclitaxel to the peritumoral brain of patients with recurrent glioblastoma with a device transplanted in the skull during surgical resection (NCT04528680). Pharmacokinetic analysis from the trial indicates that operation of the device to activate circulating microbubbles increased the concentration of albumin‐bound paclitaxel in sonicated portions of the by a factor of 3 to 4, and carboplatin by a factor of 5 to 9.^314^ These effects were achieved with no observed treatment‐related neurological deficits, and closing of the BBB over the course of an hour following the procedure.

Clinical results of BBB opening reflect conservative exposure parameters (eg, pulsing parameters to minimize BBB disruption), with no reported serious adverse outcomes. Pre‐clinical evidence encourages this caution. Disruption of the BBB can be accomplished across a wide range of exposure frequencies, peak negative pressures, pulse durations, and microbubble formulations. Off‐target bioeffects have been observed at the upper range of peak negative pressures and microbubble concentrations investigated, including red blood cell extravasation, dilated blood vessels, astroglia scars, upregulation in the transcription of proinflammatory cytokines and chemokines, glial cell activation, macrophage infiltration, and modulation of microglial activities.^202^ A mild and temporary inflammatory response was shown to promote the removal of cellular debris.^315^ The relevance of this finding is uncertain given the use of an increased microbubble concentration of approximately 5‐ to 10‐fold increased relative to other pre‐clinical or clinical studies.^313^, ^316^

Confirmation of safe exposure parameters can be achieved with MRI to determine the duration and spatial extent of BBB opening, and identify adverse events such as extravasation or bleeding.^317^, ^318^ To account for changes in the in situ acoustic field given the heterogeneity of the skull, patient‐specific simulations can be performed based on pre‐treatment computerized tomography (CT) data.^319^ Real‐time passive cavitation detection is also being investigated to gauge the efficacy and safety of BBB procedures.^316^, ^320^

In addition to enhancing drug delivery, focused ultrasound‐induced BBB disruption can be used for diagnostic purposes. Liquid biopsy has entered clinical practice to guide treatment for numerous tumor types,^321^ but remains challenging for brain cancers due to the BBB.^322^ The use of focused ultrasound to permeabilize the BBB is thought to enable “2‐way trafficking,” both to enhance drug delivery as well as increase the prevalence of tumor‐derived biomarkers in the bloodstream.^323^ Further, focused ultrasound provides spatiotemporal control to determine the location of suspicious lesions. This sonobiopsy method improves the detection of glioblastoma mutations in murine and porcine models^324^, ^325^ A first‐in‐human sonobiopsy trial with neuronavigation delivery to apply focused ultrasound indicated an enrichment in plasma circulating DNA in patients with high‐grade glioma.^326^ Cavitation monitoring was used to mitigate any inertial activity,^327^ and there was no observable tissue damage in this trial.

Similar to the BBB, the BSCB prevents dissemination of therapeutics from the vasculature.^328^ This barrier is problematic for the 5% of patients with solid tumors that develop leptomeningeal metastases.^329^ Inspired by work on BBB disruption, investigators have developed methods to improve the delivery of therapeutics through BSCB via focused ultrasound‐induced microbubble activity. Initial studies in a rodent model of leptomeningeal metastases demonstrated good suppression of tumor growth when trastuzumab was combined with focused ultrasound and microbubbles. Findings indicated that the tumor volume was reduced for the focused ultrasound group relative to trastuzumab alone.^330^ A particular challenge applying focused ultrasound to the BSCB is bone that surrounds the target, which cause standing waves that decrease targeting accuracy.^331^ To overcome this challenge, specialized dual aperture sources that use short burst, phase keying exposures have been proposed to mitigate standing waves.^332^ This approach has been shown to be successful for penetrating the BSCB in rodents and swine.^302^, ^333^

### Sonothrombolysis

Ultrasound has been explored as a means to expedite dissolution of a thrombus for the treatment of stroke, pulmonary embolism, myocardial infarction, and venous thrombosis.^334^ Depending on the exposure conditions, ultrasound can act either as a standalone therapy, or as adjuvant to thrombolytic drugs. Acoustic cavitation is a primary mechanism of action to enhance outcomes for sonothrombolysis therapies.^335^ Stable cavitation can act as a micro‐pump to enhance the penetration of thrombolytic drugs into the thrombus while removing fibrin degradation products.^336^ Minimal changes have been observed in the clot burden for stable cavitation alone, demonstrating that thrombolytics are a necessary component for this treatment strategy.^337^ The ability of stable cavitation to enhance a thrombolytic drug depends on the clot subtype.^338^ Stable cavitation effectively enhances the action of thrombolytics for unretracted, porous clots. These outcomes enable a dose reduction of thrombolytic drugs,^339^ a key requirement to mitigate bleeding complications associated with thrombolytic therapy. Thrombolytic therapy is not improved via stable cavitation in clots characteristic of subacute or chronic disease. These aged thrombus subtypes are more resistant to thrombolytic therapy^340^ but may be prone to inertial cavitation.^341^ Indeed, sonothrombolysis has been demonstrated to be effective in acute thrombus for histotripsy or histotripsy‐like exposures and retracted thrombus.^134^, ^342^, ^343^, ^344^, ^345^

Multiple devices have been explored for sonothrombolysis, ranging from focused transducers to imaging probes with extended acoustic outputs.^346^, ^347^, ^348^ Transcranial systems developed to treat stroke include unfocused, low‐frequency systems for rapid treatment based on landmark‐based targeting,^349^, ^350^, ^351^ and MRI‐guided focused transducers.^352^ Venous thromboemboli have been treated with intravascular and extracorporeal systems. The EKOS endovascular system is used for pulmonary embolisms that are surrounded by bone and air‐filled tissue.^353^ The system was cleared by the U.S. FDA in 2005, though this clearance did not include coadministration of a cavitation nucleation agent. Newer intravascular systems have been developed with an intention to co‐deliver nanometer‐sized droplets as bubble nucleation agents.^354^, ^355^ Extracorporeal systems are primarily developed for targeting deep vein thrombosis in the iliofemoral vasculature. Both spherically and cylindrically focused transducers have been produced for rapid targeting through electronic steering along the length of the vessel.^347^, ^348^

Outcomes for clinical testing of sonothrombolysis techniques have been mixed. The “CLOTBUST‐ER” trial (NCT01098981) found no difference at 90 days for stroke patients treated with standard‐of‐care thrombolytic alone with or without 2‐MHz transcranial ultrasound exposure.^356^ The trial ended early because outcomes were not improved relative to the administration of thrombolytic alone. There were no issues in terms of patient safety, consistent with metanalysis of prior studies that used ultrasound to enhance thrombolytic drugs.^357^ It was hypothesized that the hands‐free device designed for this study provided inadequate coverage of the thrombus. Similar outcomes were observed in the Norwegian Sonothrombolysis in Acute Stroke Study (NOR‐SASS),^358^ which included the addition of a microbubble agent. High mechanical index diagnostic ultrasound pulses (5‐μs duration, MI = 1.1–1.5, 1.8 MHz) in combination with microbubbles have been shown to restore epicardial and microvascular flow in acute ST‐segment elevation myocardial infarction (NCT02410330).^359^ Patients who received ultrasound showed an improvement in terms of resolution of the ST‐segment and infarct volume relative to percutaneous coronary intervention alone. These results suggest there is a role for sonothrombolysis, though technological developments are needed to ensure effective and safe microbubble activation.

### Sonodynamic Therapy

The known capacity of inertial cavitation to produce rare chemical species has been investigated as a means to provide therapeutic benefit.^360^ Photosensitive agents that generate reactive oxygen species following exposure to light have been studied for over a century,^361^ including to saturate tumors with cytotoxic chemicals (ie, photodynamic therapy). Sonodynamic therapy sought to provide an acoustic analog to photodynamic therapy,^362^, ^363^ capitalizing on the fact that several photosensitizing agents can also be activated by ultrasound.^364^ Unlike light, ultrasound can be applied to targets several centimeters deep, giving sonodynamic therapy a distinct advantage relative to photodynamic therapy.^365^ Common sonosensitizing agents include titanium dioxide,^366^ Rose Bengal,^367^ PpIX,^368^ and fluorescein.^369^ The inclusion of 5‐aminolevulinic acid (5‐ALA) increases the uptake of sonosensitizers like PpIX in proliferating cells such as glioma, thereby disrupting the heme pathway to promote apoptosis following ultrasound exposure.^370^, ^371^, ^372^

The precise mechanism of activation for sonodynamic therapy remains under investigation,^373^ though light generation during inertial cavitation remains a common theme.^374^ To accentuate cavitation effects, several groups aim to develop moieties to co‐deliver bubble nuclei with sonodynamic agents.^287^, ^367^ There are significant differences in gas content between microbubble contrast agents (eg, perfluorocarbon) and early studies into bubble‐induced light emissions (eg, air with a need for noble gasses).^375^ Nevertheless, photons have been detected from ultrasound activation of microbubbles in vitro and in vivo.^376^, ^377^ Despite their lack of noble gasses, oxygen‐loaded microbubbles have been shown to generate sufficient light to activate the sonodynamic agents.^376^ These findings demonstrate there is still a lack of understanding of the primary mechanisms of the process for light generation during cavitation. Given that a primary mechanism for sonodynamic therapy is the formation of reactive oxygen species, the therapy has been shown to have poor outcomes in hypoxic tumors that lack molecular oxygen.^378^ Hypoxia is a ubiquitous eventual outcome for all solid tumors.^379^ To ensure efficacy for sonodynamic therapy, oxygen‐loaded microbubbles have been developed to titrate the targeted tissue to a normoxic state.^380^

There are several clinical trials that have tested sonodynamic therapy. A cohort of 3 patients with metastatic breast cancer were treated using a combination of sonodynamic and photodynamic therapy.^381^ The targeted tumors displayed good regression, though patients experienced pain as the result of treatment. In a study with 115 patients, various cancer diagnoses were treated with sonodynamic and photodynamic therapy.^382^ Outcomes with the combination approach were favorable, with an increase in median survival time and no adverse events. Positive outcomes were linked to an acute inflammatory response that activated the innate immune system. Additional trials are underway to test sonodynamic therapy for neuro‐oncology applications (NCT06039709, NCT04845919, NCT04559685, NCT05362409, NCT05370508, and NCT05123534).

### Sonoporation

Ultrasound has been shown to accelerate drug and gene transfection for a number of years.^383^ Compared with other transfection methods, sonoporation provides the best potential for clinical adoption due to its high specificity, deep tissue penetration, and low cost.^384^ The generation of transient pores in cells via cavitation is hypothesized to be the primary mechanism of action for sonoporation.^385^ Hence, microbubble contrast agents are a critical component for any sonoporation strategy. The precise mechanisms of pore formation are under investigation. Stable cavitation generates microstreaming that increases shear stresses near the cell wall,^386^ which has been associated with membrane pore generation.^385^ Inertial cavitation effects may also contribute, including shock waves or jetting from an asymmetric collapse.^387^, ^388^, ^389^ The observed duration of permeabilization ranges from a few seconds up to 24 hours.^390^ In some cases, the pore may be permanent. For example, 1‐MHz pulses of 10 cycles and 0.85‐MPa peak negative pressure were applied to microbubbles affixed to MRC‐5 fetal fibroblasts.^391^ Openings less than 3 μm in diameter resealed the cell membrane, but pores larger than ~6 μm diameter persisted indefinitely. Beyond the physical mechanisms responsible for pore generation, the resulting increased penetration of molecules into the cell cytoplasm is also not fully understood. Hypotheses range from non‐selective poration of the cell membrane, endocytosis stimulation, and large cell membrane wounds.^390^

There have been few updates to clinical adoption of sonoporation since 2012, though several new applications have emerged. Multiple studies have investigated sonoporation for mRNA and DNA vaccines delivery.^392^, ^393^ To facilitate vaccine delivery, designer microbubbles were developed with mRNA incorporated into the lipid shell.^394^ Ultrasound‐induced microbubbles activity enhanced transfection by more than a factor of 40 relative to intramuscular injection, resulting in modification of genetic expression for more than 400 days.^393^ Sonoporation methods have been developed to load the cryoprotectant trehalose into erythrocytes, enabling storage of the formed blood elements at room temperature. The resulting product is a “freeze‐dried blood” for transfusions. More than 95% of erythrocytes can be recovered following sonoporation, and ~90% after rehydration.^395^ Microfluidics devices are under development to perform sonoporation to accentuate trehalose transfection.^396^

### Cavitation‐Enabled Therapy for Hypertrophic Cardiomyopathy

Hypertrophic cardiomyopathy is a life‐threatening condition associated with progressive heart malfunction, which can lead to sudden death in otherwise healthy young athletes.^397^ Available treatment methods are invasive, including intra‐cardiac surgery and alcohol ablation.^398^, ^399^ Inertial cavitation can reduce excessive heart muscle to address hypertrophic cardiomyopathy. Proof‐of‐concept for this approach was demonstrated in a rodent model using ultrasound pulses of 1.5 MHz fundamental frequency and 4 MPa peak negative pressure. These exposure conditions resulted in good cavitation, as visualized on B‐mode grayscale with a 10 MHz imaging probe.^400^, ^401^ Therapeutic efficacy was indicated by premature ventricular contractions induced by the cardiomyocyte injury, and measurements of troponin.^402^ Ultrasound pulses were ECG‐gated to coincide with the end of systole, which resulted in the best production of premature complexes with minimal heart function disruption.^403^ The resulting effect was tissue reduction indicated on vital staining.^404^ The lesions healed within ~6 weeks with minor fibrin formation but effective heart function.^405^ Application of this approach on a genetic rat model of hypertrophic cardiomyopathy resulted in a 16% reduction in endocardial wall thickness, which was considered a clinically significant outcome.^406^

An undesirable consequence of the treatment was swelling in heavily treated volumes which led to infarction‐like injury. Swelling associated with these off‐target effects was substantially reduced by steroids, and cardiac fibrosis limited by losartan. The prevalence of excessive injury was reduced when the fractionating therapy occurred over the course of 3 treatment sessions separated by 1 week.^407^ These results demonstrate the promise of cavitation‐enabled tissue reduction for hypertrophic cardiomyopathy, though additional tests are required prior to the initiation of a large‐scale clinical study.

### Radiosensitization

Therapeutic ultrasound in combination with microbubbles has been shown to increase the sensitivity of tumors to radiation therapy. Disruption of the BBB with ultrasound combined with systemic microbubbles increases regional blood flow and oxygenation.^408^ These conditions are necessary for the formation of free radical by radiation, and the resultant DNA damage.^379^ Radiotherapy combined with ultrasound‐induced BBB opening was found to enhance tumor control and prolonged survival compared to radiotherapy alone in a murine model of glioblastoma.^409^ These results led to a prospective pilot study for patients with recurrent high‐grade glioma that included reirradiation for disease control (NCT04988750). A BBB opening procedure was performed 2 hours prior to fractioned stereotactic radiosurgery or conventional radiotherapy. Analysis of the initial 6 patients indicated there were no BBB opening‐related adverse events. One patient had a partial response after the combined procedure or an objective response rate of 16.7%.

Inertial cavitation also increases tumor sensitization to radiation therapy,^410^ potentially via vascular damage.^411^, ^412^, ^413^, ^414^, ^415^, ^416^, ^417^ Ultrasound‐activated microbubbles damage endothelial cells and induce thrombosis, similar to outcomes of high‐dose radiation. Acidic sphingomyelinase (ASMase) activation within the endothelium results in ceramide‐induced cell death and rapid apoptosis.^413^, ^417^, ^418^, ^419^, ^420^ This process enhances low (less than 6 Gy) and high (more than 8 Gy) radiation doses to provide a synergistic approach to cancer treatment.^412^, ^413^, ^416^, ^417^, ^419^, ^421^, ^422^ These results indicate endothelial cell death due to vascular shutdown and direct canonical DNA damage are primary contributors to ultrasound‐enhanced radiotherapy.

Insights from preclinical studies on ultrasound vascular ablation have led to several clinical trials. A pilot clinical trial (NCT03199274) explored ultrasound‐triggered microbubble destruction to enhance the treatment response in hepatocellular carcinoma patients undergoing transarterial radioembolization.^423^ Ultrasound pulses with a 1.5 MHz fundamental frequency, 2.3 μs duration, and mechanical index of 1.13 were applied at a rate of 100 Hz for several minutes prior to radioembolization. A higher tumor response rate was noted in participants who received microbubble destruction compared to those who received radioembolization alone. No significant complications were reported in this study. A first‐in‐human, phase 1 prospective interventional trial (NCT04431674) of 8 breast cancer patients assessed fractionated radiotherapy combined with focused ultrasound‐microbubble treatment (800 kHz fundamental frequency, 570 kPa peak negative pressure, and 16‐cycle tone burst over a 50 ms period repeated for 5–10 minutes). The ultrasound microbubble treatment was applied an hour prior to the first and fifth radiotherapy fractions.^424^ Seven patients experienced a complete response at the treated site over 3 or more months of follow‐up. There were no late effects related to radiotherapy or toxicity from the ultrasound‐microbubble treatment, indicating a significant potential for radiosensitization. These results have prompted another clinical trial underway to test ultrasound microbubble destruction to radiosensitize head and neck cancers (NCT04431648).

### Mechanical Ablation

Histotripsy relies on peak negative pressures in excess of 25 MPa to generate spontaneous bubbles for tissue ablation (see Histotripsy section).^126^ Such pressures can be difficult to achieve in situ due to factors such as aberration and complex acoustic paths.^425^, ^426^, ^427^ To mitigate these issues, nanodroplets have been explored to lower the threshold for bubble nucleation. Formulations of nanodroplets to target micro‐metastases were developed and tested in vitro.^428^ Initial studies found a significant decrease in the threshold for bubble cloud formation with ~200 nm diameter nanodroplets (~10 MPa) compared to without the droplets (~28 MPa) for 500‐kHz pulses of 2 cycle duration.^228^ The droplet transition threshold was found to increase with frequency for single‐cycle pulses,^429^ in contrast to measurements with non‐histotripsy insonations.^242^ These results suggest homogenous nucleation of the perfluorocarbon liquid under histotripsy,^429^ whereas heating via superharmonic focusing is a mechanism for non‐histotripsy droplet transition.^257^, ^430^ A larger number of pulses was required for complete treatment with nanodroplets relative to insonation without droplets, indicating exogenous nuclei lower the efficiency of histotripsy relative to intrinsic nuclei. Other nuclei for histotripsy have also been investigated, including nanocones and microbubbles.^226^, ^431^, ^432^ Nanobubbles combined with low frequency, low amplitude ultrasound (~80 kHz fundamental frequency, ~250 kPa peak negative pressure) have been shown to produce mechanical ablation in vitro and in vivo.^282^, ^433^, ^434^

## Monitoring Bioeffects

**Figure 4:**
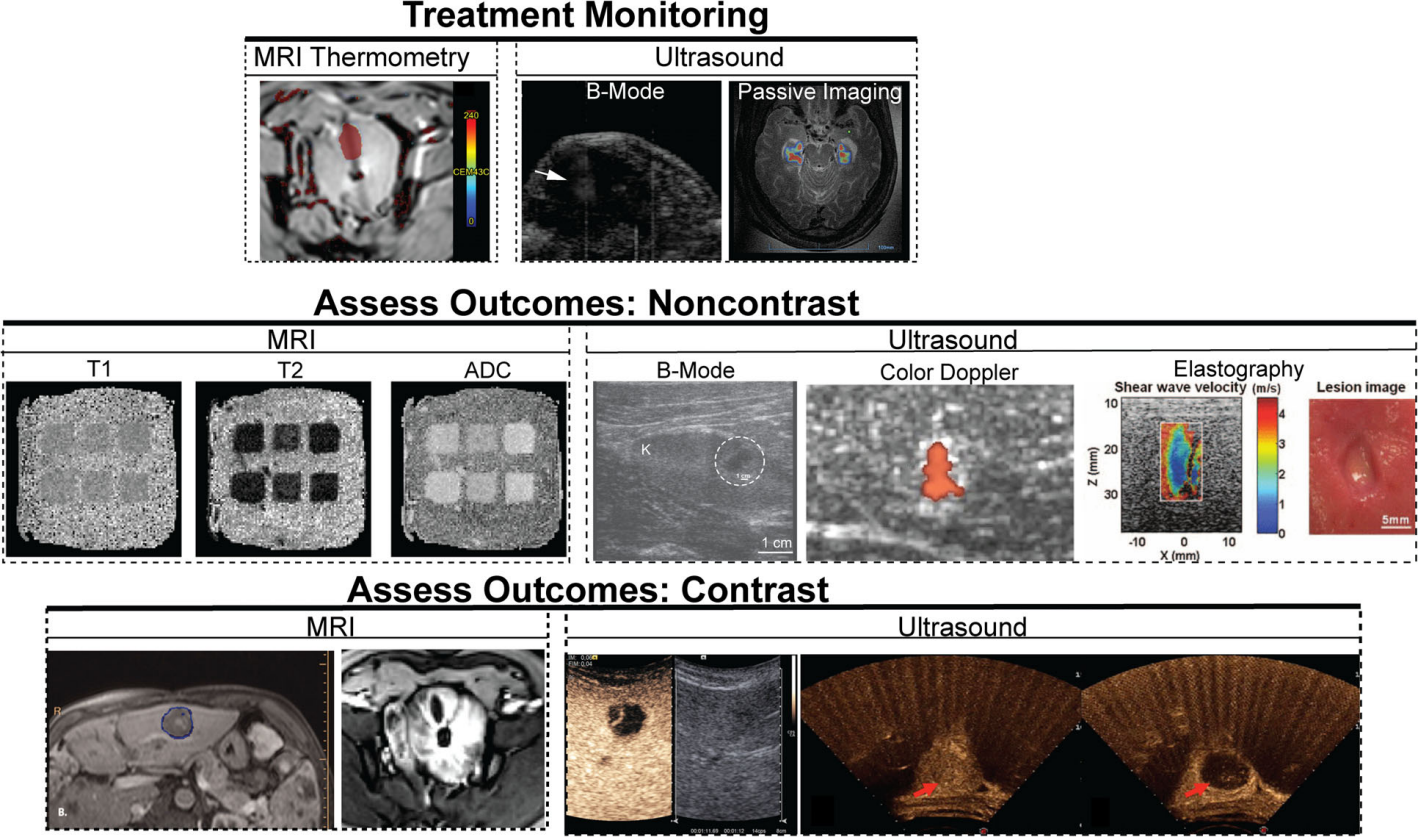
Imaging for treatment monitoring and assessment of outcomes is critical for successful therapeutic ultrasound. *Treatment monitoring*: Temperature is tracked with MRI thermometry (Reprinted from *Medical Physics*, vol. 46, no. 2, MRI‐guided transurethral insonation of silica‐shell phase‐shift emulsions in the prostate with an advanced navigation platform, pp. 774–788, Copyright 2019, with permission from Wiley). Cavitation can be gauged by hyperechogenicity on B‐mode imaging or the detection of acoustic emissions (Reprinted from *Brain*, vol. 146, no. 3, Blood–brain barrier opening of the default mode network in Alzheimer's disease with magnetic resonance‐guided focused ultrasound, pp. 865–872, Copyright 2023, by permission of Oxford University Press). *Noncontrast assessment of outcomes*: Native MRI contrast (Reprinted from *Physics in Medicine and Biology*, vol. 62, no. 17, The response of MRI contrast parameters in in vitro tissues and tissue mimicking phantoms to fractionation by histotripsy, article number 7167, Copywrite 2017, with permission from IOP Publishing), standard ultrasound grayscale (Reprinted from *Scientific Reports*, vol. 9, Pilot in vivo studies on transcutaneous boiling histotripsy in porcine liver and kidney, article number 20176, Copyright 2019, with permission from Nature Publishing under creative commons license CC BY 4.0), bubble‐induced color Doppler (Reprinted from *IEEE Transactions on Ultrasonics, Ferroelectrics, and Frequency Control*, vol. 63, no. 6, Bubble‐induced color Doppler feedback for histotripsy tissue fractionation, pp. 408–419, Copyright 2016, with permission from IEEE), or elastography (Reprinted from *IEEE Transactions on Ultrasonics, Ferroelectrics, and Frequency Control*, vol. 59, no. 6, Imaging feedback of histotripsy treatments using ultrasound shear wave elastography, pp. 1167–1181, Copyright 2012, with permission from IEEE) can be used to gauge treatment outcomes. *Contrast assessment of outcomes*: Regions of nonperfusion on contrast MRI or ultrasound are primary methods used to assess ablation (Reprinted from *International Journal of Hyperthermia*, 39(1), First‐in‐man histotripsy of hepatic tumors: the THERESA trial, a feasibility study, pp. 1115–1123, Copyright 2022, with permission from Taylor and Francis Group under creative commons license CC BY 4.0; Reprinted from *Medical Physics*, vol. 46, no. 2, MRI‐guided transurethral insonation of silica‐shell phase‐shift emulsions in the prostate with an advanced navigation platform, pp. 774–788, Copyright 2019, with permission from Wiley; Reprinted from *IEEE Transactions on Ultrasonics, Ferroelectrics, and Frequency Control*, vol. 68, no. 9, Contrast‐Enhanced Ultrasound: A Useful Tool to Study and Monitor Hepatic Tumors Treated With Histotripsy, pp. 2853–2860, Copyright 2021, with permission from IEEE; Reprinted from *European Journal of Radiology*, vol. 81, no. 12, Clinical Utility of a Microbubble‐Enhancing Contrast (“SonoVue”) in Treatment of Uterine Fibroids with High Intensity Ultrasound: A Retrospective Study, pp. 3832–3838, Copyright 2012, with permission from Elsevier).

Therapeutic ultrasound is a non‐ or minimally invasive procedure. To ensure the procedure is accurate and effective, imaging is used for therapy planning, monitoring (eg, track heating or cavitation), and assess outcomes. The type of imaging will vary based on the pathologic target, therapeutic mechanism, and system (Figure 4). This section will focus on imaging associated with bioeffects in the therapy monitoring and assessment of outcome stages. Imaging for pre‐treatment planning has been described previously.^435^

### Ultrasound Imaging

#### Treatment Monitoring

Focused ultrasound systems are often fit with a coaxial imaging probe. The type of ultrasound imaging used for treatment monitoring depends on the intended bioeffect. Bubbles are effective scatters of ultrasound, making even a standard B‐mode imaging sequence a starting point to track cavitation‐based therapies.^436^ Imaging sequences intended for diagnostic use have limitations for therapy monitoring, including the presence of interference between the imaging and therapy pulses. These artifacts can limit meaningful assessment during application of the therapy. Modified imaging sequences have been proposed to address constructive interference, including filtering to remove the fundamental therapeutic frequencies and the corresponding harmonics or pulse inversion.^437^, ^438^ Ultrafast, plane wave sequences enable the imaging and therapy sequences to be interleaved, thereby avoiding interference.^439^ Scatter from cavitation can be limited with plane waves due to their unfocused nature, particularly for targets at depth. The integration of a chirp‐coded excitation sequence has been shown to significantly increase bubble detection.^440^ Additional processing of the steered signals with a nonlinear Volterra filter increases the bubble‐to‐contrast ratio more than 20 dB relative to standard image processing.^441^ Ultrafast pulses interact with cavitation to enhance the rate of bubble dissolution,^442^ a rate‐limiting factor for cavitation‐based histotripsy.^443^

Diagnostic imaging systems can also be used to monitor ultrasound therapy through the detection of acoustic emissions (eg, cavitation). Structures within tissue scatter the incident therapy pulse, and can be used to determine in situ information about shifts in focal location due to aberration.^444^, ^445^ Cavitation is often the strongest source of acoustic emissions and is among the primary considerations when monitoring bubble‐based therapies. Indeed, acoustic emissions have been shown to correlate with a number of bioeffects, such as ablation,^439^, ^446^, ^447^ drug delivery,^134^, ^337^ and BBB or BSCB disruption.^448^, ^449^, ^450^, ^451^ Historically, emissions were detected with single element transducers. The received signals were processed to indicate the strength of spectral components associated with inertial (eg, broadband or inharmonic emissions) or stable (eg, harmonics,^452^ subharmonics,^316^ or ultraharmonics^336^) cavitation. Studies have also used imaging probes as passive detectors.^453^, ^454^ The received signals are processed in an analogous method to standard B‐mode sequences to form images that not only quantify the strength of acoustic emissions, but their location and extent. These images are termed passive acoustic maps or passive cavitation images, and have been an active field of research since the early 2010s.^444^, ^453^, ^454^, ^455^, ^456^ With the advent of commercial ultrasound imaging systems operated with high‐level computing languages, passive imaging has become more readily integrated into pre‐clinical studies.^457^ Passive imaging has been implemented onto clinical‐grade scanners,^458^ and tested in a trial for delivery of temperature‐sensitive liposomes in combination with cavitation nuclei (ISRCTN17598292) and neuronavigated BBB opening with microbubble contrast agents (NCT04118764).

Passive images are often collected without using absolute time of flight information, which restricts resolution based on the point spread function of the array.^457^ For standard imaging probes, the point spread function is increased by a factor of 10 along the range dimension relative to the axial dimension for delay‐and‐sum‐like beamformers. This means there is poor localization of cavitation along the central axis of therapy source for co‐axially placed imaging probes. Outcomes are improved for custom detection arrays. For instance, transcranial systems have a point spread function that spans approximately 1 wavelength in all directions.^459^ A number of approaches have been proposed to address the resolution of passive imaging,^460^, ^461^, ^462^ which demonstrates this is a primary area of research for the field of therapeutic ultrasound. Due to variability in detection instruments, there has been significant interest in the development of absolute, system‐independent measurements of cavitation.^463^ Methods to calibrate imaging systems to report system‐independent data have been outlined, including accounting for effects such as diffraction,^464^ attenuation,^465^ and probe configuration.^466^

The use of ultrasound for monitoring thermal therapies has also been investigated, largely through the collection of quantitative acoustic parameters. Ultrasound imaging‐based temperature mapping can be achieved based on assessment in shifts of the speed of sound.^467^, ^468^, ^469^ These estimates of tissue heating have not shown the same level of robustness as MRI thermometry. Other quantitative ultrasound parameters have been shown to be good surrogates for heating over temperatures from 38 to 64°C, such as the effective acoustic concentration or scatterer diameter.^470^

#### Treatment Outcomes

Ultrasound imaging is often the initial approach used to gauge treatment outcomes. For standard B‐mode imaging, thermal ablation appears hyperechoic due to an increase in perfusion and boiling bubbles generated within the focal zone.^471^, ^472^ Changes in echogenicity have been found to correlate with tissue temperature.^473^ This information was integrated onto a transrectal prostate ablation device to guide the acoustic output based on changes in echogenicity. Outcomes for 97 patients treated with echogenicity feedback resulted in a post‐treatment negative biopsy rate of 97%.^99^ Ultrasound images processed with echo decorrelation are less sensitive to artifacts and more sensitive to ablation than classical quantitative acoustic metrics.^474^ Based on its success in pre‐clinical studies, echo decorrelation was tested in a clinical study for radiofrequency ablation.^475^ Histotripsy ablation reduces tissue structure to acellular debris, resulting in a hypoechoic appearance on B‐mode imaging for *ex vivo* specimen and phantoms.^341^, ^476^, ^477^ The appearance of histotripsy ablation can be more difficult to assess in vivo,^140^ prompting the development of a neural network for image segmentation of ablation.^478^ The network performed well in vitro, with an overall accuracy of 82%, IoU score of 92%, and area under the ROC curve of 92%. The network had poor predictive ability in the early stages of treatment due to morphological changes in the appearance of ablation on ultrasound imaging.

Other non‐contrast modes of ultrasound imaging have been investigated to assess outcomes. Ablation disrupts the microvasculature, which appears as a reduction in blood flow under power and color Doppler imaging.^479^ Changes in the color Doppler are also indicative of successful tissue fraction under histotripsy due to changes in bubble motion.^480^, ^481^ Ablation alters the elastic properties of the tissue. Thermal coagulation increases tissue stiffness,^482^ which can be tracked with ultrasound shear wave elastography.^483^ Advanced elastography sequences take advantage of the therapy pulse to generate shear waves.^484^ In contrast, histotripsy liquefaction reduces tissue stiffness. Complete treatment generates liquefaction that prohibits the generation of shear waves, and therefore produces a null region on elastography images.^485^

Contrast‐enhanced imaging provides a proxy for perfusion within the tissue, particularly for intravascular microbubble contrast agents.^32^ Both thermal and mechanical ablation disrupt the capillary bed, which prevents the penetration of contrast into treated regions. The resulting ablation gives a hypointense appearance on contrast‐enhanced ultrasound.^140^, ^486^, ^487^, ^488^ There can be some question on the margins of treatment, which can appear hyperintense due to sublethal damage.^472^ A similar hypointense rim is also observed for other forms of contrast‐enhanced imaging (eg, MRI or CT).^84^

In summary, ultrasound imaging provides real‐time information and is substantially less expensive than other modalities.^489^ There remain limitations to the use of ultrasound imaging for therapy guidance, including inaccuracies in estimating the ablation zone extent, and interobserver and objective variability in assessment of outcomes.^490^, ^491^ Standard ultrasound scanners largely lack volumetric assessment, which is a deterrent relative to other imaging modalities.

### Magnetic Resonance Imaging

#### Therapy Monitoring with MRI Thermometry

The known temperature dependence of the proton resonance frequency shift for water is well established (on the order of −0.01 ppm/°C),^492^ and can be assessed with MRI. Successive MRI acquisitions with phase‐sensitive sequences like gradient echo enable the generation of accurate temperature change maps in near real‐time.^493^ Temperature monitoring with MRI is used extensively to guide thermal ablation, both for targeting and to gauge outcomes based on thermal dose.^18^, ^494^ There are limitations to the accuracy of MRI thermometry. Assessment of proton resonance frequency requires sufficient water content, posing a challenge to assess temperature shifts in fatty tissue such as breast or liver.^495^ There can be artifacts due to drifts in the magnetic field or high heating that cause phase wrapping.^496^ Finally, proton resonance frequency thermometry is sensitive to macroscopic motion (eg, caused by the patient moving) and even changes in blood flow.^497^

#### Treatment Outcomes

Excellent soft tissue contrast can be achieved with MRI to evaluate ablation. Even ultrasound‐guided treatments often rely on MRI to confirm outcomes 24–72 hours after the procedure.^436^ There are several forms of inherent MRI contrast to determine treatment outcomes, including longitudinal relaxation time (T1), transverse relaxation time (T2), and proton density. Diffusion is another form of MRI contrast, and is well correlated with histopathology.^498^, ^499^, ^500^, ^501^ For thermal ablation, changes in diffusivity depend on the tissue type and insonation parameters.^501^, ^502^ Mechanical ablation increases local diffusivity and perfusion within targeted regions.^499^, ^503^, ^504^ Intravoxel incoherent motion is an extension of diffusion‐weighted imaging that accounts for diffusion and perfusion of the tissue.^505^ Based on its ability to distinguish between inflammation and necrosis, intravoxel incoherent motion shows promise to help identify the edges of treatment zones.^506^ Longitudinal and transverse contrast have also been employed to assess ablation in pre‐clinical models.^427^, ^499^, ^503^, ^507^ Quantitative T2 mapping has been applied to thermal ablation of desmoid tumors, with progressive changes observed in the relaxation time over the course of treatment.^508^

Contrast‐enhanced MRI remains the gold standard to assess ablation. Non‐perfusing regions correlate strongly with histologically assessment of tissue.^84^ Contrast‐enhanced MRI is twice as sensitive to ablation relative to contrast CT.^509^ Heating can cause ambiguity in determining the precise borders of the treatment zone due to reactive hyperthermia.^510^ For histotripsy, hyperintense contrast within major vessels have been observed in the otherwise hypointense treatment zone.^436^ These findings are consistent with the known properties of histotripsy to spare major vessels.^132^ In addition to ablation, contrast MRI is used to confirm BBB and BSCB disruption studies in pre‐clinical and clinical studies.^317^, ^449^, ^511^ Good correlation has been observed between contrast enhancement and extravasated dyes in pre‐clinical studies.^302^, ^512^

There are some additional considerations when performing contrast MRI with typical gadolinium‐based agents. Contrast MRI is performed at the end of treatment due to a decrease in the half‐life of gadolinium with increasing temperature.^513^ Gadolinium cannot be cleared by patients with renal insufficiency.^514^ Recent studies indicated the agent may be deposited and retained within the brain,^515^ though there is a lack of clinical evidence that this creates off‐target effects. Finally, the increased healthcare costs for MRI relative to ultrasound are a consideration when considering the systems used for image guidance.^516^

### Computed Tomography

Hounsfield units are a relative quantitative measurement of radio density.^517^ There is temperature dependence for Hounsfield units, which make CT a potential method to monitor thermal therapies.^518^ Real‐time monitoring with CT is not common due to the associated ionizing radiation. The primary use of CT is to assess ablation outcomes. Similar to MRI, regions of ablation appear as hypointense on contrast CT acquired with a 3‐phase sequence.^436^, ^519^ There can be a rim of enhancement surrounding the primary ablation zone indicative of benign physiologic response to thermal injury. The enhancing rim is usually resolved over the course of 1 month,^520^ and the hypointense ablation zone involutes as tissue recovers from the treatment.^519^

A further use of CT is for treatment planning of transcranial or spinal procedures with multi‐element phased array sources. The approximate mapping between Hounsfield units and acoustic properties are included in calculations to estimate the in situ acoustic field.^521^ The calculation is used to develop appropriate targeting strategies to account for aberration through appropriate phasing of each element of the therapeutic source.^522^ Information about aberration collected with CT can also be used to correct passive imaging for treatment monitoring, and hence better localization of cavitation.^459^

There can be limitations with identifying lesions with the imaging probe coaxial to the therapy source, particularly for deep‐seated targets. To address this limitation, methods that use computed tomography have been developed in the pre‐treatment planning phase for histotripsy procedures.^523^ This approach uses a well‐characterized phantom to determine the transfer functions between a cone‐beam CT system and robotic arm use for placement of the histotripsy source. Once registered, the robotic positioner for the histotripsy system places the transducers within to target regions under cone‐beam CT with 1.4 ± 0.5 mm precision.

## Pre‐Clinical Testing, Simulations, and Other Non‐Clinical Considerations

### Tissue‐Mimicking Materials

Therapeutic ultrasound systems are often calibrated or evaluated with tissue‐mimicking materials (TMMs). Compared to animal and human studies, experiments with TMMs are relatively inexpensive, easier to conduct, more reproducible, and have fewer regulatory hurdles. For most applications, it is important that TMMs model the attenuation coefficient and speed of sound to approximate the propagation of ultrasound correctly. The nonlinearity coefficient should be considered for TMMs used to test high Gol'dberg number conditions,^524^ including thermal ablation,^525^ histotripsy,^526^ and lithotripsy.^143^ Quantitative acoustic parameters (eg, backscatter coefficient) should be properly addressed to evaluate an ultrasound‐guided system.^470^ For MRI‐guided therapies, the longitudinal relaxation time (T1) and effective spin–spin‐relaxation time (T2*) should be replicated.^527^, ^528^

#### Phantoms for Thermal Therapies

Thermal properties of a TMM will impact the spatial distribution of heating, and therefore the prediction of thermal bioeffects. Phantoms have been developed that approximate the soft tissue thermal properties such as conductivity, diffusivity, and specific heat.^525^, ^528^, ^529^, ^530^, ^531^, ^532^, ^533^ Some of these TMMs also mimic the backscatter coefficient,^525^, ^530^, ^532^ nonlinearity coefficient,^525^, ^530^ or mechanical properties.^526^, ^528^, ^529^, ^530^, ^532^ The acoustic,^525^, ^529^, ^534^, ^535^ thermal,^525^, ^529^, ^536^, ^537^ and mechanical^529^ properties of TMMs have been measured over a range of temperatures relevant for thermal therapies (ie, room temperature to 70°C). To qualitatively assess thermal therapies, a polyacrylamide hydrogel and bovine serum albumin TMM was developed that becomes opaque in locations heated to 60°C.^534^, ^535^, ^538^, ^539^ Thermochromic ink‐based TMMs also provide visual cues for outcomes of therapeutic levels of heating.^540^, ^541^

Targets other than soft tissue can by modeled by phantoms. Blood TMMs within the physiologic range of attenuation coefficient, speed of sounds, nonlinearity coefficient, thermal conductivity, thermal diffusivity, and viscosity have been produced.^532^, ^536^, ^537^, ^542^ The temperature‐dependent properties of 2 blood phantoms were characterized up to 70°C.^536^, ^537^ A 2‐component phantom that approximates bone within soft tissue was generated to test palliation of metastases with MRI‐guided focused ultrasound.^543^ A bilayer gel TMM was developed to study phase aberration that causes defocusing of the acoustic field across multiple tissue layers.^544^

#### Phantoms for Histotripsy

Mechanical ablation with histotripsy relies on bubble activity to lyse cells in the focal zone.^545^ The most common TMM used in pre‐clinical studies to evaluate histotripsy is an agarose gel that includes a thin layer of red blood cells.^477^ Over the course of histotripsy exposure, treated regions become optically transparent and serve as a ground truth for identifying ablation. Further, changes in the phantom appearance on ultrasound imaging are consistent with the appearance of tissue ablated *ex vivo*. Fiducial markers can be embedded in the phantom to facilitate registration between digital photographs of ablation and medical imaging (eg, ultrasound or MRI).^499^ Agarose has a low attenuation coefficient relative to soft tissue, which may alter nonlinearities in the histotripsy pressure waveform relative to that generated in vivo.^526^ Additives such as evaporated milk increase the TMM attenuation within the range of *ex vivo* tissues, though at a loss of transparency.^526^ While the concentration of agarose can be altered to adjust outcomes based on material stiffness,^132^, ^341^ there are questions as to whether this material truly replicates the viscoelastic properties of tissue that affect bubble dynamics in vivo. More recent work with hydrogels has been shown to replicate tissue toughness, particularly replicating structures with significant extracellular matrix such as benign prostate hyperplasia.^546^ Additional phantoms with multiple layers to approximate soft tissue aberration have also been investigated.^547^

### Quality Assurance

Clinical‐focused ultrasound systems are tested on a regular basis to identify possible malfunctions prior to patient treatment. Several publications document quality assurance (QA) protocols for single‐institution studies.^548^, ^549^, ^550^ A report by the American Association of Physicists in Medicine (AAPM) Task Group 241 includes recommendations for periodic testing of MRI‐guided focused ultrasound systems.^551^, ^552^ The recommendations are divided into categories according to the frequency of inspection. Tests that are conducted daily or before each patient treatment include transducer focusing capability, transducer steering, imaging signal‐to‐noise ratio, safety interlock evaluation, visual check of equipment for damage, and inspection of the coupling membrane. Tests to be conducted every 6 months or every twenty patients include motor system evaluation, table positioning and homing capability, the accuracy of MRI thermometry, planning/delivery software function evaluation, cavitation detection, and degassing system. Tests that should be conducted annually or every 100 patients include acoustic output power with a radiation force balance and ultrasound beam characterization with a hydrophone.^551^

The AAPM Task Group 333 was formed in 2019 to develop a more detailed MRI‐guided focused ultrasound systems QA protocol that should be executed before each patient treatment. Further, the protocol was tested in an interlaboratory comparison study. The protocol included specifications for a new phantom that was custom‐designed for MRI‐guided focused ultrasound systems. Detailed methods for measurements of peak temperature rise, targeting error, signal‐to‐noise ratio, and assessing the thermal ablation volume were included as part of data acquisition required for the protocol. Five institutes were included in the interlaboratory comparison study, each with a variety of commercial and custom therapy systems. The study was completed in 2023, and processing of collected data was underway at the time this manuscript was written (June 2024).

### Reporting Therapeutic Ultrasound Treatment Parameters

Recommendations have been developed to document therapeutic ultrasound studies to promote experimental reproducibility.^553^ There are separate sets of recommendations for clinical and preclinical studies to provide flexibility for differences in expertise and equipment among institutions. The reporting recommendations include descriptions of driving electronics, transducer, hydrophone‐based acoustic output characterization, numerical simulations, monitoring methods, and the intended bioeffect.

**Figure 5:**
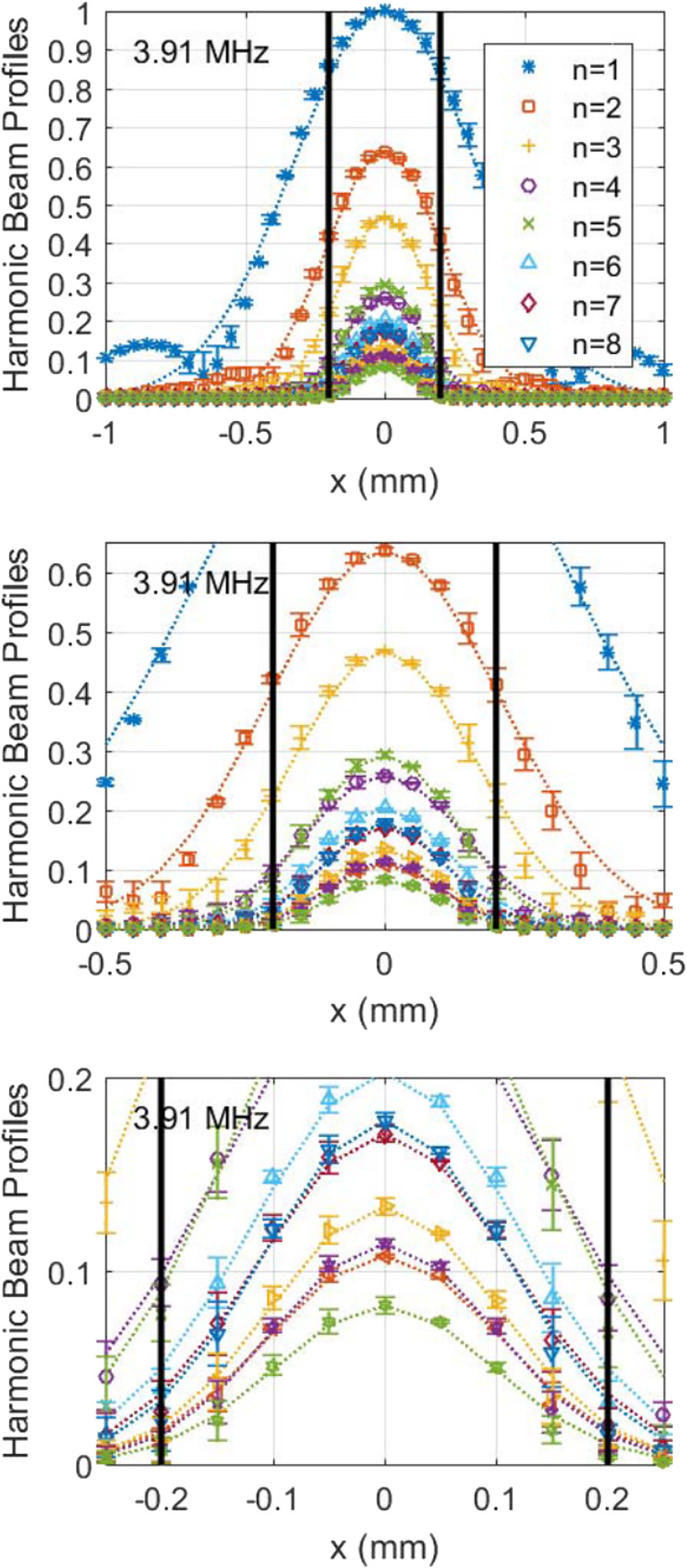
Harmonic beam profile plots for nonlinear pressure waves produced by a focused therapy transducer measured with a high‐resolution (100‐μm diameter geometrical sensitive element) fiber‐optic hydrophone. The harmonic number is denoted by *n*. Gaussian fits are shown in dotted lines. Error bars denote standard deviation in the measurement obtained from transverse scans obtained horizontally and vertically. The black vertical lines show the spatial extent of a medium‐resolution (400‐μm diameter geometrical sensitive element) low‐cost, robust needle hydrophone. The 400‐μm hydrophone can be used for accurate measurement of the acoustic field after spatial averaging correction is applied. Each panel is the same dataset but at differing extensions along the spatial dimension (Reprinted from *IEEE Transactions on Ultrasonics, Ferroelectrics, and Frequency Control*, vol. 66, no. 9, Correction for Spatial Averaging Artifacts in Hydrophone Measurements of High‐Intensity Therapeutic Ultrasound: An Inverse Filter Approach, pp. 1453–1464, Copyright 2019, with permission from IEEE). BBB, blood–brain barrier; FUS, focused ultrasound.

Measurements of the acoustic field with a hydrophone remain a mainstay parameter needed to ensure the reproducibility of therapeutic ultrasound studies. The effective size of a hydrophone element can be much larger than its geometric size, particularly at low frequencies.^554^ Methods have been developed to compensate for spatial averaging across the hydrophone sensitive element and for hydrophone frequency‐dependent sensitivity,^555^ including for broadband therapeutic ultrasound pressure pulses.^555^, ^556^, ^557^, ^558^, ^559^ The broadband nature of therapeutic ultrasound pulses is due to nonlinear propagation, resulting in energy at the fundamental frequency of the excitation (*f*_0_) and harmonic frequencies (*nf*_0_, where *n* = 1,2,3, …*n*_max_, where *n*_max_ can exceed 100).^130^, ^560^ The −3 dB beamwidths of the harmonic beam components of the therapeutic ultrasound acoustic field decrease with *n*. Critical values of *n* can exist for an excitation with harmonic beamwidths smaller than the sensitive element of the hydrophone (Figure 5). The resulting effect is equivalent to the application of a low pass filter to pressure waveforms recorded by the hydrophone. To address the need for spatial averaging correction, an inverse‐filter method has been shown to improve the accuracy for hydrophone measurements of focused ultrasound pressure fields.^561^ This method was adopted into an International Electrotechnical Commission (IEC) standard.^562^ To facilitate implementation of the spatial averaging correction, a simplified method was developed that is equivalent to the full model.^563^, ^564^

### Numerical Simulations

The acoustic output for operational driving conditions of therapeutic sources can be sufficient to either damage hydrophones or cause inaccurate measurements due to cavitation or excessive heating. Numerical simulations have been employed to address the gap in field measurements. Modeling may provide a better estimate of the field than measurements for some conditions due to limitations in the hydrophone bandwidth or spatial averaging effects.^130^ The IEC has released technical specification TS62556 to provide guidelines in the use of numerical calculations to report a subset of field parameters for therapeutic ultrasound devices.^565^, ^566^, ^567^, ^568^

There are multiple open‐source codes available to calculate the linear,^569^ nonlinear,^570^, ^571^ and shocked^560^, ^572^ acoustic fields and pressure waveforms of ultrasound sources (Table 2). Some codes also calculate Pennes bioheat transfer equation in parallel.^566^, ^573^, ^574^ These codes can often be run with a high‐level computing language (eg, MATLAB) to facilitate ease of use. The assumptions integrated into calculations for each model should be considered when analyzing data.^575^ For example, sources that are highly focused (eg, *f*‐number less than 1.2) require additional considerations to address diffraction correctly.^572^ Increased computational power may be required in the form of workstations or clusters for highly nonlinear waveforms that include shock waves.^576^ Differences among models to predict acoustic fields are unknown. The development of benchmarks for numerical computations is a topic being reviewed by IEC Technical Committee 87.

**Table 2:** Summary of Open‐Source Software Packages for Computing the Acoustic Fields of Biomedical Devices

Code	Platform	Wave Equation	Solution Method	Exposure Duration	Medium	Heating Calculation	Reference
Texas KZK	Fortran	KZK	FDTD	CW	Homogenous	No	Lee, 1995
K‐Wave	MATLAB/ C++	Westervelt	k‐space pseudospectral	CW or PW	Heterogenous (Arbitrary)	Yes	Treeby, 2012
FOCUS	MATLAB	KZK	Fast Nearfield/Angular Spectrum	CW or PW	Homogenous	Yes	Chen, 2008
Field II	C/MATLAB/Python	Linear	Tupholme‐Stepanishen	CW	Homogenous	No	Jensen, 1992
HIFU Simulator	MATLAB	KZK	Split‐Step^+^	CW	Heterogenous (Layered)	Yes	Soneson, 2009
HITU Simulator	MATLAB	Wide Angle KZK	Split‐Step^+^	CW	Heterogenous (Layered)	Yes	Soneson, 2017
HIFU Beam Simulator	MATLAB	KZK or Westervelt	Fractional step^+^	CW or PW	Heterogenous (Layered)	Yes	Yuldashev, 2021
mSOUND	MATLAB	Westervelt	Mixed‐Domain	CW or PW	Heterogenous (Arbitrary)	No	Gu, 2021

For nonlinear solvers, there can be significant error in the Khokhlova‐Zabolotshaya‐Kuznetsov (KZK) calculation if the source has an f number less than 1.2 unless the wide‐angle parabolic approximation is applied.

CW, continuous wave; PW, pulsed wave; +, code is computationally efficient for shock waves.

### Patient Safety

Precautions for MRI‐guided focused ultrasound procedures have been reviewed previously at length.^551^ Briefly, therapeutic ultrasound transducers can potentially apply excessive heat to superficial tissues between the source and the intended target.^551^, ^577^, ^578^ This problem can be ameliorated by cycling the transducer power to allow for adequate cooling between exposures,^579^ circulating cooling water,^580^ or using cooling balloons.^581^, ^582^

Sub‐therapeutic exposures can be performed to verify the accuracy of targeting. Extra care should be taken to avoid collateral exposure to nearby tissues that are thermally sensitive, such as nerves, bones, and bowel. Cavitation detection should be used to minimize the risk of undesired mechanical bioeffects.^551^

The standard to promote patient safety for therapeutic ultrasound procedures (IEC 60601‐2‐62) does not allow: 1) display of incorrect numerical values associated with the therapy; 2) production of unwanted ultrasound output; 3) production of excessive ultrasound output; 4) reflection of excessive ultrasonic power at the transducer/patient interface due to inadequate coupling; 5) unwanted targeting of tissue regions away from the intended target region; and 6) production of unwanted thermal or mechanical tissue damage in or distal to the region of interest.

## Challenges for Therapeutic Ultrasound

Despite its promise, challenges remain for therapeutic ultrasound prior to adoption into routine clinic use. The ultrasound field does not penetrate gas bodies such as the lung or the bowel. These sites represent the second and third most common cancers locations in the United States, with an estimated annual patient population of ~350,000.^583^ Lung flooding with an isotonic solution has been used successfully for pulmonary ultrasound imaging,^584^ and was tested for targeting nodules with focused ultrasound.^585^ Successful outcomes were achieved with *ex vivo* tissue human specimen, and in vivo porcine lung with no reported complications. A tumor in the anterior portion of the lung was treated with thermal ablation.^586^ No flooding was used for the treatment of this patient with favorable outcomes. Outcomes were favorable, but there has been no subsequent follow‐up to the treatment of additional patients. Targeting the pancreas is also complicated by overlapping gas‐filled intestine. Pre‐clinical studies in a swine orthotopic model pre‐treated animals with simethicone and bisacodyle to minimize the contents of the intestines.^587^ Partial ablation with histotripsy was generated in the targeted pancreatic tumors, in part due to the dense stroma. Gas was still present in the gastrointestinal tract which limited targeting, suggesting additional methods to empty intestines may be beneficial.

Defocusing of the acoustic field due to aberration is a challenge for targets like the kidney, liver,^85^ and brain.^588^ Investigators are working to address these issues with multi‐element systems the adjust the phase of the output to correct for aberration.^85^, ^425^ Simulations based on computed tomography imaging of the skull have been used to estimate the degree of aberration, and appropriating phasing of the elements.^589^ Pulses of ~1 MHz from specialized elements integrated in the array are used to localize the skull. These data are co‐registered with CT imaging, which may enable transcranial targeting without the need for MRI.^590^, ^591^ Pre‐clinical investigations into transcranial histotripsy use information collected from acoustic emissions generated by cavitation in addition to CT imaging data to correct for aberration. This 2‐step protocol achieved ~90% recovery of the peak focal pressure compared to gold standard hydrophone correction on 3 excised human calvaria.^592^ Further, there was a substantial reduction in the error of positioning the focal zone within the intended target relative to other approaches.

Off‐target ablation may occur as the result of respiratory motion.^593^ Longitudinal studies in a rodent model indicate tissue ablated by histotripsy resolves over the course of several days,^427^ suggesting that collateral damage from mechanical ablation does not have lasting effects. In contrast, thermal ablation scars treated tissue indefinitely,^594^, ^595^ which motivates the development of methods to minimize off‐target damage. Using the fact that tissue motion is along 1 primary direction, elliptical treatment plans have been tested.^596^ The resulting ablated region aligned well with the intended spherical target. Therapeutic transducers may be affixed to advanced robotic systems to translate the focal volume throughout the intended target.^597^, ^598^ Information on target motion based on ultrasound imaging has been used to adjust the robot position, and therefore therapeutic source, in real time. Robotic‐based motion‐based correction has been shown to reduce the targeting errors by 89% relative to a stationary source.^599^

The perfusion of targets can influence the outcome of focused ultrasound. Heat sink effects in highly perfused tumors diminish the efficacy of thermal‐focused ultrasound procedures.^600^ Investigations are underway to address heat sink effects, including liquid perfluorocarbon droplets and fibers.^601^, ^602^ Focused ultrasound procedures that rely on heating can be several hours in duration.^603^, ^604^ This is in part due to nearfield heating, which distorts the acoustic field and can lead to collateral damage. Nearfield heating causes skin burns at low rates of incidents,^605^ though long pauses are integrated into the treatment profile to avoid these effects.^578^ Methods are needed to avoid bones and air cavities in the near or far field that could cause irregular or unintended heating to expedite thermal ablation procedures.

Refinement is also needed for image guidance. To align the focal region with a given target, co‐registered ultrasound imaging probes used for treatment monitoring are often offset several centimeters from target. The resulting target is in the far field of the imaging system, which causes a reduction in image quality. Novel sequences or therapy‐specific imaging probes may help to address this issue. Cavitation emissions are a common approach to monitor bubble‐based therapies, but appropriate quantification and correlation with bioeffects have yet to be fully established. A virtual workshop was held in 2021 to establish the state of the field for bubble detection and quantification. Information gathered from that workshop is being processed to provide recommendations to the community when reporting on the detection of cavitation.^606^ MRI remains the gold standard for temperature quantification, though has limitations in terms of accessibility and healthcare costs.^607^ To address these issues for transcranial applications, neuronavigation is being investigated to retain the benefits of pre‐treatment diagnostic scans without the need for MRI during ultrasound exposure.^326^, ^608^, ^609^

## Conclusions

There have been substantial shifts in the landscape of therapeutic ultrasound in the years since the parent to this review article was published in 2012.^5^ An additional 23 therapeutic ultrasound devices were given FDA clearance or approval (Table 1). Treatment for 14 conditions with therapeutic ultrasound is now reimbursable through insurance, and routine clinical treatment was approved for an additional 18 conditions.^82^ The expanded use of new devices into the clinical will determine which systems are adopted into the standard‐of‐care, and potentially provide new information on bioeffects in therapeutic ultrasound.

The emergence of new applications of therapeutic ultrasound outlined in this review largely reflects recent advances in medicine. Some examples include investigations into the immunomodulatory effects of therapeutic ultrasound, which are consistent with the rise of checkpoint inhibitors into the clinic.^610^ The neuromodulatory effects of transcranial ultrasound align with the recognition that improved approaches are needed to address mental health.^611^ Sonogenetics complements gene editing techniques like CRISPR‐Cas9.^612^ Mechanotransduction has been identified as an increasingly important tool in biology, lending into the need for approaches with targeted microbubbles or other cavitation nuclei to provide localized, controlled perturbation.^613^ The development of new therapeutic ultrasound techniques is likely to reflect future challenges in healthcare, which may be the result of multiple factors.

Based on the information outlined in this review, the following should be considered for pre‐clinical and clinical tests of therapeutic ultrasound technology:

- Although clinically relevant bioeffects have not been observed when ultrasound is used by qualified professionals for diagnostic imaging and image‐guided intervention, sufficient and controlled exposure levels can be used to generate therapeutic benefits.
- The type of bioeffect generated by ultrasound depends on many factors, including the ultrasound source, exposure conditions, presence of cavitation nuclei, and tissue type.
- Appropriate monitoring techniques based on the mechanism‐of‐action and intended bioeffect should be implemented.

## Data Availability

Data sharing is not applicable to this article as no new data were created or analyzed in this study.
